# FIRRM cooperates with FIGNL1 to promote RAD51 disassembly during DNA repair

**DOI:** 10.1126/sciadv.adf4082

**Published:** 2023-08-09

**Authors:** Edgar Pinedo-Carpio, Julien Dessapt, Adèle Beneyton, Lauralicia Sacre, Marie-Anne Bérubé, Romain Villot, Elise G. Lavoie, Yan Coulombe, Andréanne Blondeau, Jonathan Boulais, Abba Malina, Vincent M. Luo, Anna-Maria Lazaratos, Jean-François Côté, Frédérick A. Mallette, Alba Guarné, Jean-Yves Masson, Amélie Fradet-Turcotte, Alexandre Orthwein

**Affiliations:** ^1^ Lady Davis Institute for Medical Research, Segal Cancer Centre, Jewish General Hospital, 3755 Chemin de la Côte-Sainte-Catherine, Montréal, QC H3T 1E2, Canada.; ^2^ Division of Experimental Medicine, McGill University, Montréal, QC H4A 3J1, Canada.; ^3^ CHU de Québec Research Center-Université Laval (L’Hôtel-Dieu de Québec), Laval University Cancer Research Center, Québec, QC G1R 3S3, Canada.; ^4^ Department of Molecular Biology, Medical Biochemistry and Pathology, Université Laval, Québec, QC G1V 0A6, Canada.; ^5^ Department of Biochemistry, McGill University, Montréal, QC H3G 0B1, Canada.; ^6^ Département de Biochimie et Médecine Moléculaire, Université de Montréal, Montréal, QC H3C 3J7, Canada.; ^7^ Maisonneuve-Rosemont Hospital Research Centre, Montréal, QC H1T 2M4 Canada.; ^8^ Montreal Clinical Research Institute (IRCM), Montreal, QC H2W 1R7, Canada.; ^9^ Department of Microbiology and Immunology, McGill University, Montréal, QC H3A 2B4, Canada.; ^10^ Département de Médecine, Université de Montréal, Montréal, QC H3C 3J7 Canada.; ^11^ Department of Radiation Oncology, Winship Cancer Institute, Emory University, Atlanta, GA 30322, USA.; ^12^ Gerald Bronfman Department of Oncology, McGill University, Montréal, QC H4A 3T2, Canada.

## Abstract

Interstrand DNA cross-links (ICLs) represent complex lesions that compromise genomic stability. Several pathways have been involved in ICL repair, but the extent of factors involved in the resolution of ICL-induced DNA double-strand breaks (DSBs) remains poorly defined. Using CRISPR-based genomics, we identified FIGNL1 interacting regulator of recombination and mitosis (FIRRM) as a sensitizer of the ICL-inducing agent mafosfamide. Mechanistically, we showed that FIRRM, like its interactor Fidgetin like 1 (FIGNL1), contributes to the resolution of RAD51 foci at ICL-induced DSBs. While the stability of FIGNL1 and FIRRM is interdependent, expression of a mutant of FIRRM (∆WCF), which stabilizes the protein in the absence of FIGNL1, allows the resolution of RAD51 foci and cell survival, suggesting that FIRRM has FIGNL1-independent function during DNA repair. In line with this model, FIRRM binds preferentially single-stranded DNA in vitro, raising the possibility that it directly contributes to RAD51 disassembly by interacting with DNA. Together, our findings establish FIRRM as a promoting factor of ICL repair.

## Teaser

FIRRM plays a role in the maintenance of genome stability.

## INTRODUCTION

Interstrand DNA cross-links (ICLs) are highly cytotoxic DNA lesions that produce physical obstacles for vital biological processes, including DNA replication, transcription, and recombination. ICLs can emerge from endogenous reactive aldehydes but more often arise from exposure to either naturally occurring compounds [e.g., mitomycin C (MMC)] or chemically synthesized drugs, such as cisplatin or cyclophosphamide [reviewed in (*1*)]. These ICL-inducing drugs are widely used as chemotherapeutic agents in the treatment of both solid tumors and blood cancers (*2*). Aside from compromising essential biological processes, ICLs, if left unrepaired, result in various genomic abnormalities, ranging from point mutations to chromosome breakage or missegregation and mitotic catastrophe [reviewed in (*3*)].

Each type of ICL-inducing agents leads to different types of intra- and interstrand cross-links that distort the double helix to different levels (*1*). To detect, signal, and repair these lesions, cells rely on a complex response that typically gets triggered when the replication machinery encounters the ICL and involves the precise and coordinated effort of multiples pathways, including the Fanconi anemia (FA), nucleotide excision repair (NER), and the homologous recombination (HR) repair pathways, alongside the ATR serine/threonine kinase (ATR) checkpoint signaling pathways (*1*). ICLs are initially recognized by the FA complementation group M (FANCM) complex (FANCM, FAAP24, MHF1, and MHF2) that serves as a platform for the docking of seven additional FANC proteins (FANCA-C, FANCE-G, and FANCL) as well as two additional associated proteins (FAAP20 and FAAP100) that altogether form the FA core complex. Along with FANCT (UBE2T), the E3-ubiquitin ligase FA core complex promotes the monoubiquitylation of the effector FANCI/FANCD2 (ID2) heterodimer, a critical step in the processing and subsequent repair of the ICLs. Once assembled and activated at the lesion, the ID2 heterodimer recruits structure-specific nucleases that promote ICL unhooking and acts as a molecular hub for the recruitment of translesion synthesis (TLS) DNA polymerases, which bypass the ICL adduct, and the subsequent recruitment of HR factors involved in the repair of the double-strand breaks (DSBs) generated during this process (*1*). The past few years witnessed the discovery of an important decision point during the detection and early processing of ICL, named the ICL repair pathway choice. This decision is made between replication-independent and replication-dependent pathways and appears to be dictated by the structure and the location of the ICL as well as the phase of the cell cycle in which it is detected (*1*). Recent dissection of the replication-dependent ICL repair pathway has identified a series of recently identified players such as TRAF interacting protein (TRAIP) (*4*, *5*), nei like DNA glycosylase 3 (NEIL3) (*6*), and suppressor of cancer cell invasion (SCAI)-REV3 like, DNA directed polymerase zeta catalytic subunit (REV3) (*7*, *8*) that act before the TLS and HR pathways.

Aside from a better understanding of ICL repair pathway choice, several reports have highlighted the complexity of the final stages of ICL repair, such as the resolution of RAD51-mediated strand invasion. For instance, the AAA^+^ adenosine triphosphatase (ATPase) FIGNL1 has been shown to bind to RAD51 and promote its unloading from DNA (*9*), a function that is limited by the SWSAP1-SWS1-SPIDR complex (*9*, *10*). More recently, the helicase HELQ (*11*) and the HROB-MCM8-MCM9 (*12*) complex have been shown to contribute to parallel pathways that promote DNA repair synthesis following D-loop formation. However, the spatiotemporal regulation of these functions is still unknown.

Although the first model of ICL repair was drawn two decades ago, a complete picture of the factors involved in regulating this repair pathway remains largely unclear. Thus, we performed a series of CRISPR-based genome-wide screens with the ICL-inducing agent mafosfamide (MAF), and we identified FIGNL1 interacting regulator of recombination and mitosis (FIRRM) as a player in the response to ICLs. In-depth characterization delineated its contribution to regulating the later steps of HR pathway. Proximal mapping of FIRRM proximity interactors coupled to structure-function analysis identified the AAA^+^ ATPase Fidgetin like 1 (FIGNL1) as a partner of FIRRM, an interaction that is critical for the stability of both proteins. Depletion of FIRRM resulted in the persistence of RAD51 at DSBs and concomitant cell death, two phenotypes that are rescued by a mutant (FIRRM ∆WCF) that stabilizes the protein in absence of FIGNL1. Biochemical characterization of FIRRM revealed that it directly binds to single-stranded DNA (ssDNA) in vitro, raising the possibility that this interaction contributes to RAD51 displacement from DNA at DNA damage sites. Our model thus defines FIRRM as an integral factor in the maintenance of genome stability by promoting RAD51 disassembly at ICL-induced DNA damage.

## RESULTS

### CRISPR screening identifies FIRRM as a modulator of ICL repair in BL cells

**Fig. 1.**
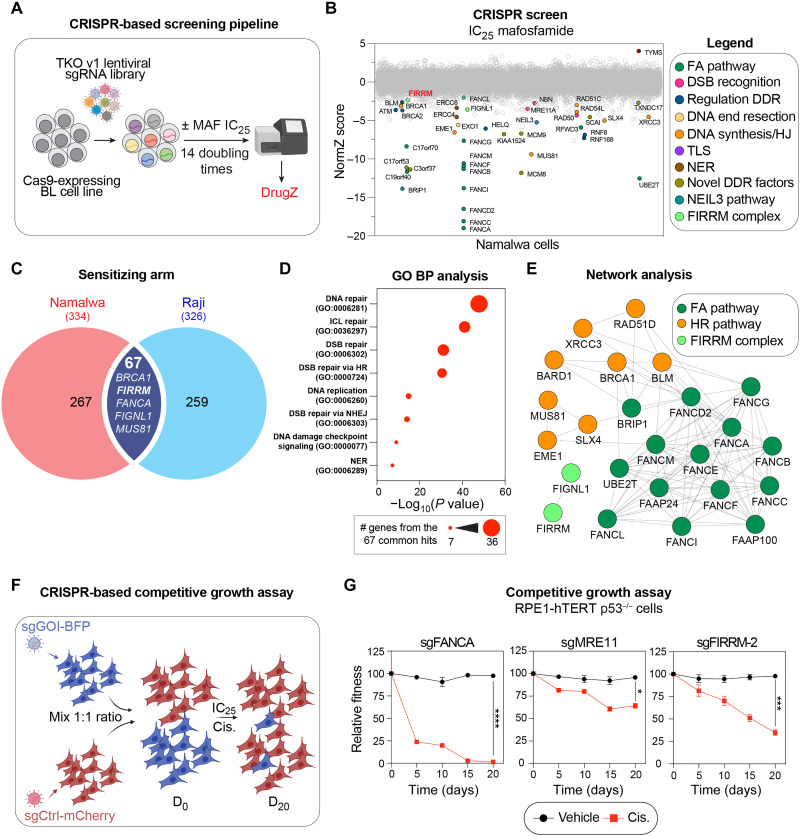
CRISPR-based genome-wide screening identifies FIRRM as a modulator of MAF sensitivity. (**A**) Schematic representation of the CRISPR-based screening pipeline used in BL cells and the screen analysis using the DrugZ algorithm. (**B**) Horizontal scatter plot of DrugZ-generated ranking of the Namalwa MAF CRISPR screen. NormZ values are plotted on the *y* axis, and gene names are plotted on the *x* axis. DDR, DNA damage response; HJ, Holliday junction. (**C**) Venn diagram displaying common sensitizing genes from Namalwa and Raji screens (for genes with NormZ ≤ 2). (**D**) Gene ontology biological processes (GO BP) diagram obtained from common sensitizing genes from Namalwa and Raji screens. GO term enrichments are ranked by statistical significance (*P* value). The size of the circles indicates the number of the 67 common gene hits within a pathway. (**E**) Network analysis displaying protein physical interactions using Cytoscape and the GeneMANIA package. The FIRRM-FIGNL1 complex is represented in the vicinity of FA and HR repair networks. (**F**) Schematic representing the pipeline used in the two-color competition assay. RPE1-hTERT p53^−/−^ cells were transduced with a sgRNA targeting either the gene of interest (GOI) coupled with BFP or LacZ gene coupled with mCherry. BFP- and mCherry-expressing cells were mixed at a 1:1 ratio at D_0_ and treated with IC_25_ cisplatin (Cis.). BFP-positive over mCherry-positive cells ratio was followed over D_20_. (**G**) Competitive growth results obtained in RPE1-hTERT p53^−/−^ cells targeted with sgRNA against *FANCA*, *MRE11*, or *FIRRM*. Cells were either treated with DMSO as a vehicle or cisplatin at its IC_25_ (2.4 μM). Mean ± SEM (*n* = 3 replicates). Data were analyzed using a one-way Welch’s analysis of variance (ANOVA) test and Dunnett’s multiple comparisons test (G). *P < 0.05, ***P < 0.001, and ****P < 0.0001.

To better understand the factors influencing ICL repair, we undertook CRISPR-Cas9 dropout screens in Namalwa and Raji human Burkitt lymphoma (BL) cell lines, using the metabolized version of the ICL agent, cyclophosphamide as a selective drug (MAF). First, we generated stable Namalwa and Raji cell lines expressing Cas9 using lentiviral transduction and confirmed genome editing efficiency by targeting the FAM83G gene as previously described (*13*). Next, CRISPR-based genome-wide screening was initiated by transducing the Toronto knock-out version 1 (TKO v1) single guide RNA (sgRNA) library in both human BL Cas9 cell lines (*14*, *15*), and transduced cells were amplified under puromycin (2 μg/ml) selection for 7 days (Fig. 1A). Both Namalwa and Raji transduced cell lines were subsequently treated with either preoptimized doses of MAF [25% inhibition concentration (IC_25_)] or with dimethyl sulfoxide (DMSO) as a vehicle for 14 doubling times (14 days for both cell lines) before being processed for next-generation sequencing (NGS). The DrugZ algorithm was used to determine the relative abundance of each sgRNA and identify genes whose knockout sensitizes cells to MAF (NormZ score < −2.5, *P* < 0.01) (Fig. 1B, fig. S1A, and table S1) (*16*). In both screens, pathway enrichment analysis identified ICL repair and HR as two biological pathways statistically enriched among our identified genes of interest (fig. S1B). As expected, most factors of the FA core complex and factors implicated in HR repair emerged as top sensitizers in both cell lines (Fig. 1B, fig. S1A, and table S1), validating our CRISPR-based screening approach to identify genes influencing ICL repair. Notably, the recently characterized *FIGNL1* (*9*, *17*), *MCM8-MCM9* (*12*), and *SCAI* (*7*) scored significantly in both of our screens (Fig. 1B, fig. S1A, and table S1).

We identified 67 target genes that function as sensitizers in both BL cell lines (Fig. 1C and table S1). Gene ontology analysis of these hits revealed enrichment for specific biological processes related to DNA repair, DNA replication, and DNA damage checkpoint (Fig. 1D and table S2), and the network constructed with these common sensitizers shows high connectivity between genes of the FA and HR pathways (Fig. 1E and fig. S1C). According to this network, we found that *FIRRM* behaves similarly to factors of the cellular response to ICL agent in BL cell lines. Previous reports identified FIRRM as a modulator of chromosome segregation through the regulation of polo like kinase 1 (PLK1) activity(*18*), and its orthologs in *Arabidopsis thaliana* (flip) and *Oryza sativa* (MEICA: meiotic chromosome association 1) have been shown to regulate meiotic recombination (*19*, *20*), thereby emerging as critical player for the maintenance of genome stability (*21*). We therefore focused our attention on FIRRM and investigated its role in the cellular response to ICL.

To validate the contribution of FIRRM in this response, we targeted it using a sgRNA in a 20-day CRISPR-based growth competition assay (*22*) that was performed in nontransformed human retinal pigmented epithelial cell line retinal pigment epithelial-1 (RPE-1) immortalized with telomerase reverse transcriptase (hTERT) (henceforth termed RPE1-hTERT p53^−/−^ cells) (Fig. 1F). Briefly, stable cell lines expressing a sgRNA of interest along with a blue fluorescent protein (BFP) reporter (sgGOI-BFP) or a control sgRNA against LacZ along with a mCherry reporter (sgCtrl-mCherry) were established. At 0 days (D_0_), the cell line expressing the sgGOI-BFP was mixed to a 1:1 ratio with the sgCtrl-mCherry cell line and treated for D_20_ with an IC_25_ of the ICL-inducing agent cisplatin (2.4 nM) before BFP and mCherry measurements at different time points. For comparison, we also tested sgRNAs that target representative genes of the FA (sg*FANCA*) and the HR pathways (sg*MRE11*). In this assay, *FIRRM* knockout hypersensitizes RPE1-hTERT p53^−/−^ cells to cisplatin (Fig. 1G and fig. S1D), demonstrating that the phenotype associated with FIRRM depletion is neither cell line nor ICL agent specific. The effect of FIRRM depletion on the cellular fitness of cisplatin-treated cells is intermediate of a core FA protein and MRE11 homolog, double strand break repair nuclease (MRE11) (Fig. 1G and fig. S1D), suggesting that FIRRM is important for cellular survival to ICL-inducing agent but may not be a core FA gene. Together, this work demonstrates that our CRISPR-based screening approach effectively identified genes affecting the response to ICL agents, including modulators like FIRRM.

### FIRRM depletion induces genomic instability and cellular senescence in a p53-dependent manner

**Fig. 2.**
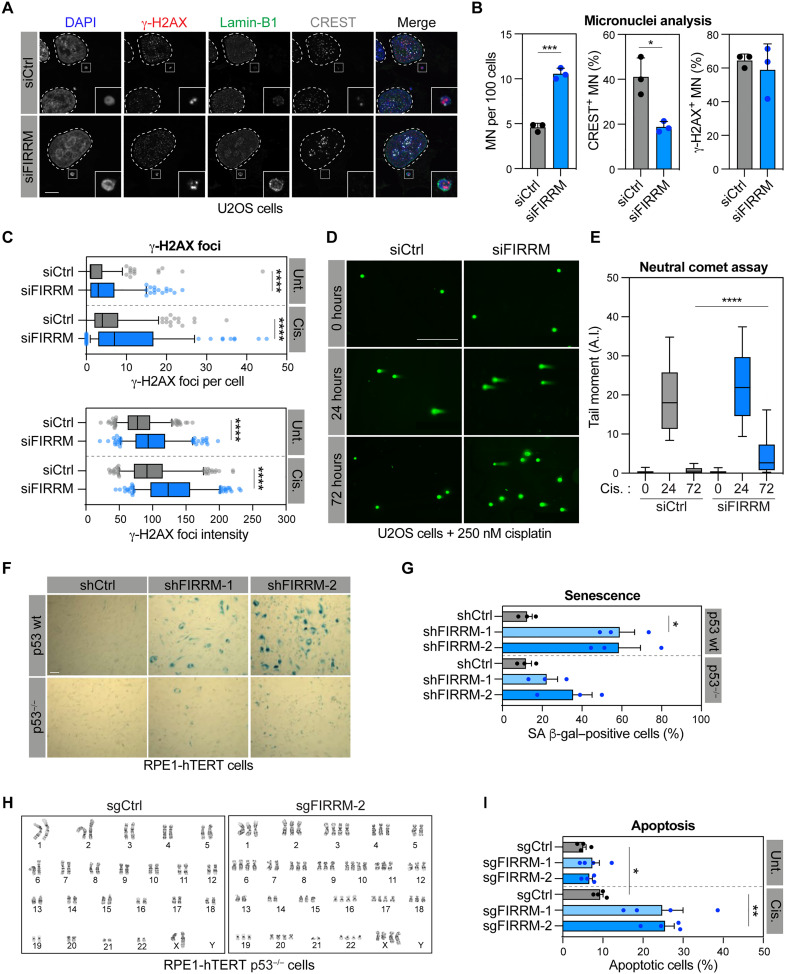
Depletion of FIRRM leads to genomic instability and senescence-induced cell death. (**A**) Representative images of U2OS cells depleted for FIRRM and processed for γ-H2AX (red), Lamin-B1 (green), and centromeres (CREST) (gray) immunofluorescence. Nuclei were counterstained with DAPI. Scale bar, 5 μm. (**B**) Quantification of cells treated as in (A). Mean ± SD (*n* = 3 independent experiments). (**C**) Quantification of γ-H2AX foci per cell (top) and γ-H2AX foci intensity (bottom) in cells depleted for FIRRM and exposed to 1 μM cisplatin or vehicle (DMSO) for 3 hours. Mean ± SD (*n* = 3 independent experiments). (**D**) Representative images of comet neutral assay in U2OS cells depleted or not for FIRRM and treated or not with 250 nM cisplatin for 24 hours and collected at the indicated time points. (**E**) Quantification of tail moment as shown in (D). Data are represented as box and whiskers (10th to 90th percentile) (*n* = 3, at least 150 cells were measured per experiment in each condition). A.I., arbitrary intensity. (**F**) Representative images of RPE1-hTERT WT and p53^−/−^ cells depleted for FIRRM and stained with SA β-gal. Scale bar, 100 μM. (**G**) Quantification of SA β-gal–positive cells as shown in (G). Mean ± SEM (*n* = 3 independent experiments). (**H**) Karyotype representation of RPE1-hTERT p53^−/−^ cells depleted (sg*FIRRM*-2) for FIRRM displaying aberrant chromosome copy numbers through metaphase analysis. (**I**) Quantification of apoptotic RPE1-hTERT p53^−/−^ cells depleted for FIRRM and treated with cisplatin. Mean ± SEM (*n* = 4, 30,000 cells measured per experiment in each condition). Data were analyzed using an unpaired *t* test with Welch’s correction (B and C), a two-way ANOVA, followed by Tukey multiple comparisons test (E) or a one-way Welch’s ANOVA test with Dunnett’s multiple comparisons test (G and I). *P < 0.05, **P < 0.01, ***P < 0.001, and ****P < 0.0001.

To define the role of FIRRM in the maintenance of genome stability, we depleted it using a small-interfering RNA (siRNA) in the human bone osteosarcoma epithelial U2OS cells (fig. S2A). At 48 hours after transfection, FIRRM-depleted U2OS cells accumulated micronuclei (MN) (Fig. 2, A and B). MN formation is triggered by unresolved genomic instability, such as DSBs and lagging chromosomes (*23*). In FIRRM-depleted cells, we observed that most MN show negative staining for centromeres but positive staining for the DSB marker phosphorylated form of the histone variant H2AX (γ-H2AX) (Fig. 2, A and B), suggesting that they arise from improperly segregated acentric chromosome fragments induced by unrepaired DSBs (*23*). 53BP1 nuclear bodies (NBs) are another marker of genomic instability, representing subnuclear structures assembled around DNA lesions caused by replication stress, and transmitted during mitosis to the daughter cells (*24*). Untreated FIRRM-depleted U2OS cells exhibit a twofold increase in the number of 53BP1-NBs in the G_1_ phase of the cell cycle (fig. S2, B and C), suggesting that FIRRM plays a role in preventing replication stress-induced DNA breaks. To further explore the impact of FIRRM on genomic stability, we quantified the number and the intensity of γ-H2AX foci that accumulate in FIRRM-depleted U2OS cells that are in interphase. We found that both the number and the intensity of γ-H2AX foci were remarkably increased in FIRRM-depleted cells treated with 1 μM cisplatin for 3 hours compared to the control (cells treated with siCtrl) (Fig. 2C and fig. S2D). Depletion of FIRRM is sufficient to increase the number and the intensity of γ-H2AX foci in untreated cells (Fig. 2C and fig. S2D), indicative of a contribution of FIRRM to protect cells from endogenous DNA damage. As a complementary approach, we quantified the dynamics of DSBs induced by cisplatin treatment (250 nM, 12 hours) in FIRRM-depleted U2OS cells using the neutral comet assay. We noted that significant levels of ICL-induced DSBs persist in FIRRM-depleted U2OS cells 72 hours after treatment compared to control conditions (Fig.2, D and E), indicative of a defect in ICL repair.

In mammalian cells, DNA damage activates checkpoints that promote cell cycle arrest, senescence, or ultimately apoptosis in a p53-dependent manner (*25*–*28*). Consistent with our previous observations that FIRRM depletion results in the accumulation of DNA breaks, we noticed that its depletion by two distinct small hairpin RNAs (shRNAs) is sufficient to induce senescence in RPE1-hTERT and IMR90 p53 wild-type (WT) cell lines as monitored by β-galactosidase (β-gal) staining and RNA levels of well-established senescent markers (Fig. 2, F and G, and fig. S2, E to I). This phenotype was significantly reduced in RPE1-hTERT cells where *TP53* was inactivated by CRISPR technology (Fig. 2, F and G). All our attempts to generate p53 WT FIRRM knockout cell lines were unsuccessful. In contrast, we could isolate two *FIRRM*^−/−^ clones in RPE1-hTERT *p53*^−/−^ that we confirmed by immunoblotting analysis (fig. S2J). Notably, karyotyping of *FIRRM* knockout RPE1 hTERT p53^−/−^ clones identified major chromosomal rearrangements, particularly duplication of chromosomes (Fig. 2H and fig. S2K). While duplication of chromosomes was only observed in 4 of 30 karyotypes in sgCtrl cells, this phenomenon is exacerbated in FIRRM-depleted clones (18 of 35 for sgFIRRM1 and 21 of 40 for sgFIRRM2), highlighting the central role of FIRRM in the maintenance of genome stability. In this context, increased levels of apoptosis detected in *FIRRM*^−/−^ cells by annexin V/propidium iodide (PI) staining showed that both clones are more prone to cell death upon cisplatin treatment (Fig. 2I and fig. S2L), confirming the protective role of FIRRM against cell death following ICL induction.

### FIRRM acts downstream of the FA pathway during ICL repair

**Fig. 3.**
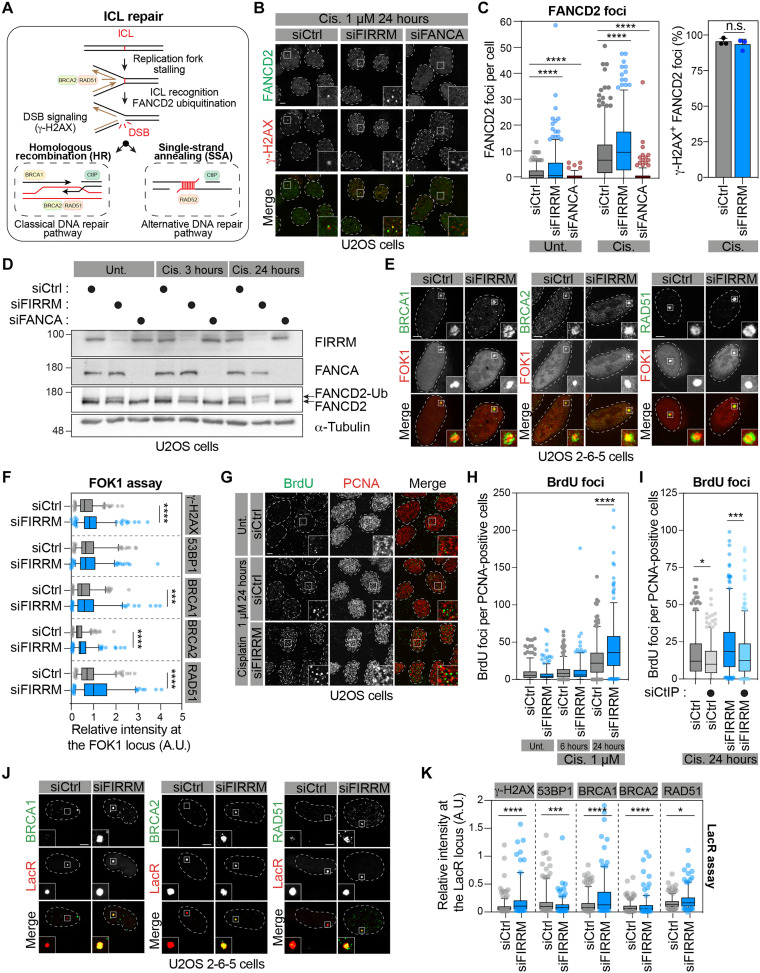
FIRRM is not a core FA gene but is required for the response to ICL and replicative stress. (**A**) Schematic of ICL repair pathways in mammalian cells. (**B**) Representative images of U2OS cells depleted for FIRRM or FANCA and treated with 1 μM cisplatin for 24 hours. Scale bar, 5 μm. (**C**) Quantification of FANCD2 foci per cell (left) and colocalizing with γ-H2AX (right) as in (B). Mean ± SD (*n* = 3, 100 cells quantified per experiment in each condition). (**D**) Whole-cell extracts (WCE) from U2OS cells depleted or not for FIRRM or FANCA and treated with cisplatin or DMSO was analyzed by immunoblot. (**E**) Representative images of U2OS 2-6-5 cells depleted for FIRRM for 48 hours and ER-mCherry-LacR-FOK1-DD expression was induced for 4 hours before fixation. Scale bar, 5 μm. (**F**) Quantification of the ratio of indicated protein foci intensity over ER-mCherry-LacR-FOK1-DD foci intensity as shown in (E). Mean ± SD (*n* = 4 independent experiments, 50 foci quantified per experiment in each condition). (**G**) Representative images of U2OS cells depleted for FIRRM and treated with 1 μM cisplatin for 24 hours and supplemented with 10 μM BrdU for 24 hours. Scale bar, 5 μm. (**H**) Quantification of data shown in (G). (**I**) Similar to (H) except that cells were depleted with siCtIP. (**J**) Representative images of U2OS 2-6-5 cells depleted for FIRRM and transfected with an empty mCherry-LacRnls plasmid for 24 hours. Scale bar, 5 μm (**K**) Quantification of data shown in (J). Mean ± SD (*n* = 3 independent experiments, 50 foci quantified per experiment in each condition). Data were analyzed using a one-way Welch’s ANOVA test and Games-Howell’s multiple comparisons test (C, left), an unpaired *t* test (C, right), or with an unpaired *t* test with Welch’s correction (F, H, I, and K). *P < 0.05, ***P < 0.001, and ****P < 0.0001. A.U., arbitrary units; n.s., not significant.

The response to ICLs relies on the activation of the FA pathway, which is induced when a replication fork encounters covalently linked DNA strands (Fig.3A), and subsequent recruitment of specific nucleases that release the ICL from one of the two parental strands (*29*). DSBs left behind by nucleolytic processing are then repaired by homology-directed recombination pathways such as HR and single-strand annealing (SSA) (Fig. 3A). To delineate at which step(s) FIRRM is participating in the ICL response, we first assessed its involvement in the recruitment and the activation of FANCD2 at ICLs. As expected, both FANCD2 accumulation and ubiquitylation at ICLs were abrogated upon depletion of FANCA in U2OS cells, serving as a control (Fig. 3, B to D). In contrast, FIRRM depletion did not impair the detection of FANCD2 at γ-H2AX foci (Fig. 3, B and C) and its mono-ubiquitylation status after cisplatin treatment (Fig. 3D). These data suggest that FIRRM plays a role downstream of the FA pathway during the response to ICLs. We noticed that FANCA-depleted cells were hypersensitive to both ICL agents MAF and cisplatin in our viability assay, unlike FIRRM-depleted cells, which displayed a much milder response to these drugs (fig. S3A). As previously observed with γ-H2AX foci (Fig. 2C and fig. S2D), we noted that more FANCD2 foci and ubiquitylated form of FANCD2 accumulate in FIRRM-depleted cells at steady state and upon cisplatin exposure (Fig. 3, B to D).

To further dissect the stage at which FIRRM influences the ICL response, we focused our attention on key HR factors and their accumulation at DSBs in the absence of FIRRM. We took advantage of a DSB reporter system in which the recruitment of these factors can be quantified at a single genomic locus. Briefly, DSBs are rapidly induced by the recruitment of the ER-mCherry-LacRnls-FOK1-DD fusion protein at a *LacO* array integrated on chromosome 1p3.6 in U2OS cells (U2OS 2-6-5 cell line) (*30*, *31*). In this assay, depletion of FIRRM correlated with an exacerbated accumulation of several HR factors at FOK1-induced DSBs, including BRCA1 DNA repair associated (BRCA1), BRCA2, and RAD51 (Fig. 3, E and F, and fig. S3, B and C). Notably, FIRRM depletion did not affect the recruitment of the nonhomologous end joining (NHEJ) factor 53BP1 (fig. S3B), suggesting that HR may be favored in the absence of FIRRM. Using native bromodeoxyuridine (BrdU) labeling (*32*, *33*), we noted a significant increase in the levels of ssDNA in FIRRM-depleted proliferating cell nuclear antigen–positive U2OS cells treated with cisplatin (Fig. 3, G and H). This phenotype is abrogated upon depletion of CTBP-interacting protein (CtIP) (Fig. 3I), reflective of increased DNA end resection and ssDNA generation in the absence of FIRRM.

To directly assess the role of FIRRM during homology-directed DNA repair, we used the well-established HeLa and U2OS direct repeats (DR)–green fluorescent protein (GFP) reporter cell lines (*34*), as well as the U2OS single-strand annealing (SA)-GFP reporter cell line (*35*), to measure DNA repair by HR and SSA, respectively. Depletion of FIRRM with four different siRNAs resulted in a significant decrease in both assays (fig. S3, D to H), particularly with siFIRRM-3 and siFIRRM-4, which results in efficient depletion of the protein as determined by immunoblotting analysis. Notably, FIRRM depletion had a much milder impact than CtIP or RAD51 depletion in both the DR- and SA-GFP assays (fig. S3, E to H), suggestive of a regulatory role rather than a core contribution of FIRRM in homology-directed DNA repair pathways. In these conditions, we did not observe any significant impact of FIRRM depletion on the cell cycle distribution of U2OS cells (fig. S3I). Beside their contribution to DNA repair, several HR factors, including BRCA1, BRCA2, and RAD51, have been shown to protect stalled replication forks from nucleolytic processing and promote their stabilization (*36*). To determine whether FIRRM participates in this process, we took advantage of the *LacO* arrays that pose an obstacle to DNA polymerase progression when bound by LacR, resulting in a stalled replication fork (*37*). In this assay, depletion of FIRRM also correlated with an exacerbated accumulation of several HR factors, including BRCA1, BRCA2, and RAD51, upon tethering of a mCherry-LacRnls fusion protein at the *LacO* arrays (Fig. 3, J and K, and fig. S3, J and K), supporting a model where FIRRM plays a role at stalled replication forks, beyond ICL repair.

Consistent with these observations, chemogenomic profiling of CRISPR screens performed in RPE1-hTERT p53^−/−^ cells with different genotoxic agents showed that FIRRM depletion impairs predominantly cell survival (NormZ < −2.3) upon exposure to agents that induce replication roadblocks (e.g., cisplatin), base alkylation [methylnitrosoguanidine (MNNG)], and oxidative damage (KBrO_3_ and H_2_O_2_) (fig. S3L) (*38*). To validate these findings, we exposed U2OS cells to a series of DNA-damaging agents and noticed that depletion of FIRRM reduced cell viability upon treatment with cisplatin, formaldehyde (FormA), MMC, hydroxyurea (HU), and the poly(ADP-ribose) polymerase inhibitor BMN673 (fig. S3M). Notably, the lack of FIRRM did not affect U2OS viability upon exposure to either ultraviolet (UV) or ionizing irradiation (IR) (fig. S3M). Because these types of DNA lesion are primarily repaired by NER and NHEJ, respectively, our data suggest that FIRRM does not participate in these repair pathways. Together, these results support a model where FIRRM is involved in both the repair of DSBs by HR and the cellular response to different replication fork roadblocks.

### FIRRM interacts with FIGNL1 and regulates RAD51 dynamics during ICL repair

**Fig. 4.**
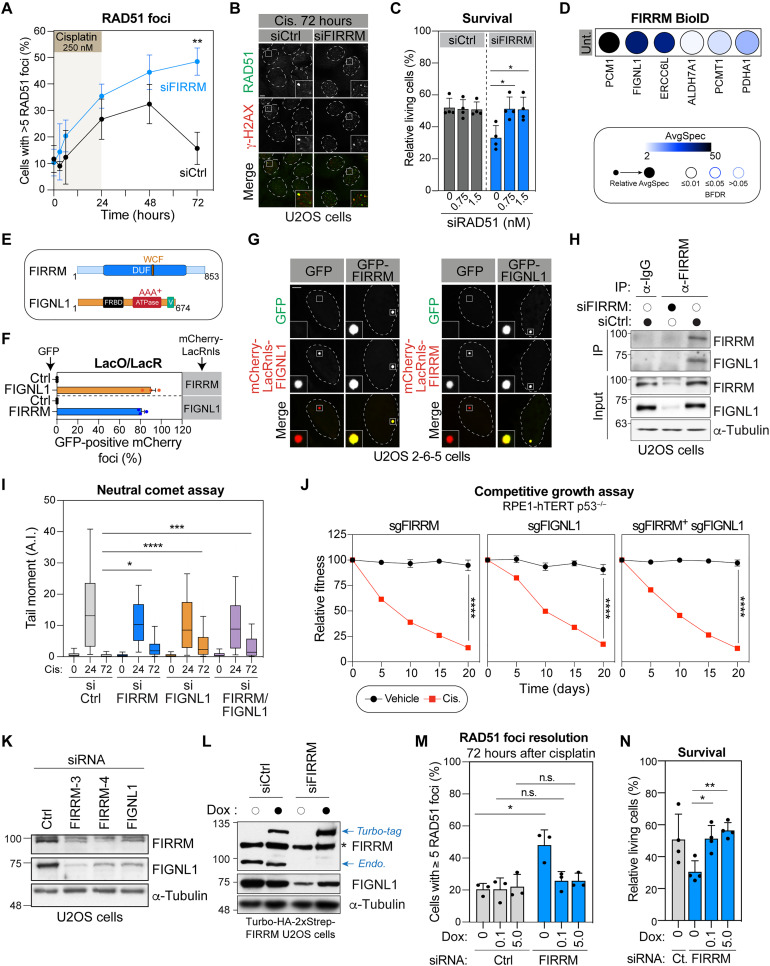
The FIRRM-FIGNL1 complex regulates RAD51 unloading from DNA. (**A**) Quantification of cisplatin-induced RAD51 foci. Mean ± SD (*n* = 3, 100 cells per experiment in each condition). (**B**) Representative images from (A). Scale bar, 5 μm. (**C**) Viability assay in U2OS cells treated with siFIRRM and siRAD51 prior cisplatin. (**D**) High-confidence proximal interactors of FIRRM identified by mTurboID. Bayesian false discovery rate, BFDR. (**E**) Schematic representation of FIRRM and FIGNL1 protein domains. DUF, domain of unknown function; WCF, WCF tripeptide sequence; FRBD, FIGNL1 RAD51 binding domain; V, vps4 domain. (**F**) Quantification of the colocalization of FIRRM and FIGNL1 at a *LacO* array. Mean ± SD (*n* = 3, 50 cells per condition). (**G**) Representative images from (F). Scale bar, 5 μm. (**H**) WCE from U2OS cells depleted for FIRRM and processed for FIRRM immunoprecipitation (IP) (*n* = 3). IgG, immunoglobulin G. (**I**) Tail moment in U2OS cells as in Fig. 2E (*n* = 3, >150 cells per condition). (**J**) Competitive growth results in FIGNL1 and/or FIRRM depleted RPE1-hTERT p53^−/−^ cells as in Fig. 1G. Mean ± SEM (*n* = 3). (**K**) WCE from U2OS cells were depleted for FIRRM or FIGNL1 and analyzed by immunoblot. (**L**) Turbo-HA-2xStrep-FIRRM U2OS cells were treated with siFIRRM and supplemented (black dot) with doxycycline (Dox). WCE was analyzed by immunoblot. * Denotes a nonspecific band. (**M**) Quantification of Turbo-HA-2xStrep-FIRRM U2OS cells with ≥5 RAD51 foci following cisplatin treatment. As in (A). Mean ± SD (*n* = 3, 100 cells for each condition). (**N**) Turbo-HA-2xStrep-FIRRM U2OS cells treated with siFIRRM, Dox, and cisplatin before survival were monitored. Mean ± SD (*n* = 4). One-way Welch’s ANOVA test and Dunnett’s multiple comparisons test for (C), (J), and (N), two-way ANOVA followed by Tukey multiple comparisons test for (I), or unpaired *t* test with Welch’s correction for (M). *P < 0.05, **P < 0.01, ***P < 0.001, and ****P < 0.0001.

To further define the role of FIRRM in DNA repair, we investigated whether its depletion affects RAD51 focus formation and resolution in U2OS cells treated with an ICL-inducing agent. As expected, RAD51 foci rapidly appeared 3 to 6 hours after the addition of cisplatin to the media (Fig. 4, A and B, and fig. S4A). Notably, more than 40% of FIRRM-depleted cells still exhibited >5 RAD51 foci 72 hours after treatment, a time point that was sufficient for the resolution of these foci in the control condition (Fig. 4, A and B). Furthermore, the intensity of the RAD51 foci in FIRRM-depleted cells was greater than the ones in cells treated with a siRNA control at a late time point (fig. S4B), suggesting that RAD51 dynamics at ICL-induced DSBs are compromised upon depletion of FIRRM. Partial depletion of RAD51 in FIRRM-depleted cells restored cell survival upon cisplatin treatment (Fig. 4C and fig. S4C), suggesting that the accumulation of RAD51 in these cells is toxic and establishing that the regulation of RAD51 dynamics by FIRRM is critical for cell survival in response to cisplatin treatment.

To gain insight into the regulation of RAD51 dynamics by FIRRM, we mapped its proximal interactome by taking advantage of the miniTurboID technology (fig. S4D) (*39*). Briefly, FIRRM was N-terminally tagged with the promiscuous biotin ligase miniTurbo (mTurboID) and stably expressed in HEK293 Flp-In cells. This construct was validated for its ability to efficiently biotinylate proximal partners by immunoblotting analysis (fig. S4E). The proximity interacting network of FIRRM was generated at steady state following 3 hours of biotin labeling. A total of 155 high-confidence FIRRM interactors found in three technical replicates were identified (table S3). Among these interactors, we identified three high-confidence candidate proteins that were also identified as sensitizers to MAF in our CRISPR screen (Fig. 4D and table S1). Two of these interactors, the pericentriolar material 1 protein and the DNA excision repair protein ERCC6-like (ERCC6L, also known as PICH), play a role in mitosis where they respectively contribute to the proper assembly of functional centrosomes and the resolution of anaphase bridges (*39*–*41*). Therefore, we reasoned that they most likely play a role in the mitotic function of FIRRM (*18*). The AAA^+^ ATPase FIGNL1 was identified as a constitutive interactor of FIRRM using mTurboID, an interaction that has also been detected previously with two orthogonal approaches [yeast two-hybrid (*19*) and affinity purification coupled to mass spectrometry (AP-MS) (*17*, *42*)]. Although FIGNL1 has been detected at centromeres, two studies support a role for the protein in regulating RAD51 filament during HR repair (*9*, *17*) raising the possibility that FIGNL1 and FIRRM act as a complex in this process.

To further characterize the interaction between FIGNL1 and FIRRM, we took advantage of the U2OS 2-6-5 cell line that contains a *LacO* array integrated into a single locus (*30*). In this context, transiently transfected mCherry-LacRnls-bait fusion proteins accumulate at the array in U2OS 2-6-5 cells, and the colocalization of preys is quantified at the mCherry locus (Fig. 4, E to G, and fig. S4, F and G). Notably, FOK1 nuclease was not expressed in this set of experiments. When fused to mCherry-LacRnls, both FIRRM and FIGNL1 were able to respectively trigger the accumulation of GFP-tagged FIGNL1 and FIRRM at the *LacO* array (Fig. 4, E to G). In contrast, neither of the mCherry-LacRnls-tagged proteins recruited GFP alone, which was used as a negative control, at the *LacO* array. Consistently, immunoprecipitation of endogenous FIRRM from U2OS cell extracts treated with benzonase confirmed that it can pull down endogenous FIGNL1 in the absence of DNA/RNA (Fig. 4H).

If FIRRM and FIGNL1 cooperate at the same stage of ICL repair, FIGNL1 depletion should result in phenotypes that are comparable to FIRRM depletion. The lack of FIGNL1 correlated with an exacerbated accumulation of several HR factors at FOK1-induced DSBs U2OS 2-6-5 cells (figs. S4, H and I), the persistence of DSBs in U2OS upon cisplatin exposure when assessed by the neutral comet assay (Fig. 4I), and a higher sensitivity to cisplatin in CRISPR-based growth competition assay (Fig. 4J). Furthermore, both siRNAs targeting FIGNL1 led to a significant defect in HR in the DR-GFP assay (fig. S4J). To test whether FIGNL1 depletion is epistatic to the loss of FIRRM, we examined the impact of either single or co-depletion of FIGNL1 and FIRRM in the neutral comet assay and the competitive growth assay upon treatment with cisplatin. In these assays, co-depletion of FIRRM and FIGNL1 did not exacerbate the phenotype observed when knocking down either of the protein (Fig. 4, I and J). Similarly, depleting FIGNL1 and FIRRM led to a similar defect in HR than FIRRM or FIGNL1 alone in the DR-GFP assay (fig. S4J). As FIGNL1 promotes RAD51 unloading at DSBs, our findings support a model where FIGNL1 and FIRRM are functionally epistatic in the resolution of DNA breaks.

During these experiments, we noticed that depletion of either FIRRM or FIGNL1 by siRNA drastically reduce the level of its respective partner (Fig. 4K), suggesting that FIRRM-FIGNL1 interaction is critical for the stabilization of the complex. Consistent with this model, we found that reexpression of FIRRM in FIRRM-depleted cells rescued the stability of FIGNL1 along with the resolution of cisplatin-induced RAD51 foci and cellular resistance to cisplatin treatment in survival assay (Fig. 4, L to N, and fig. S4, K to M). The U2OS cell line expressing an inducible cassette of TurboID-HA-2xStrep-FIRRM was obtained by infecting cells with lentivirus, selecting the transduced population with puromycin and isolating clones. We observed that the expression of the tagged version of FIRRM induces a concomitant reduction of both endogenous FIRRM and FIGNL1 at high doses of doxycycline, highlighting the codependency of their stability and revealing a tight regulation of their levels in cells (fig. S4N). The requirement for such regulation is still unclear as high levels of FIRRM by itself is not toxic for the cells (fig. S4O).

### A mutant that stabilizes FIRRM in the absence of FIGNL1 efficiently promotes RAD51 resolution at ICLs

**Fig. 5.**
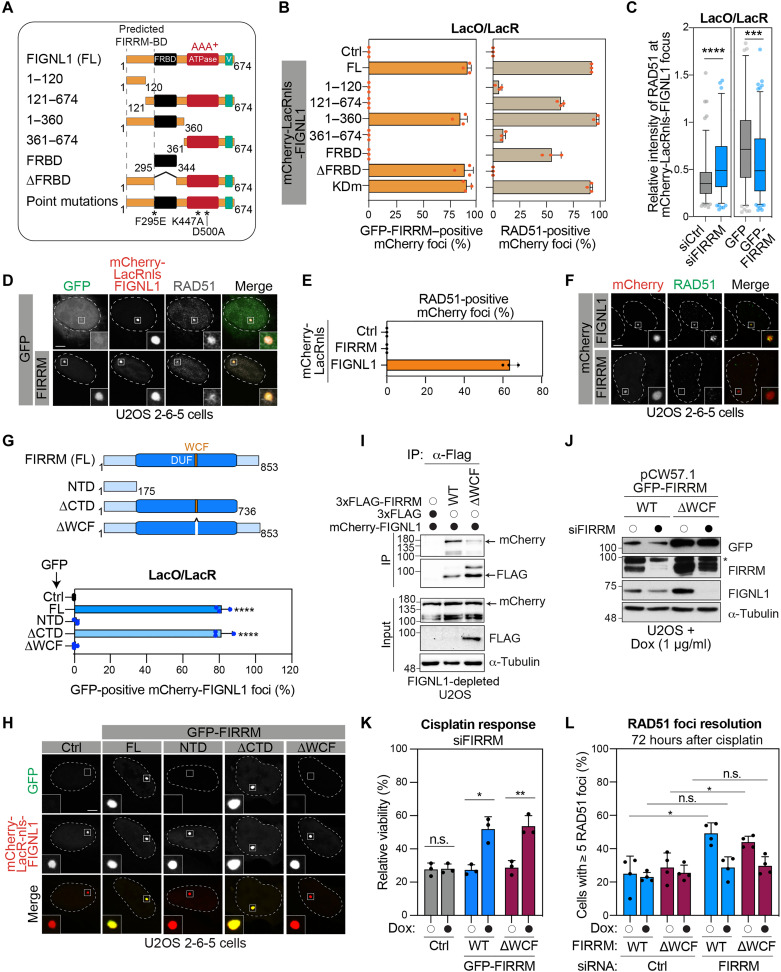
A stable FIRRM is capable of promoting RAD51 unloading. (**A**) Schematic representation of FIGNL1 constructs used in this study. (**B**) Quantification of mCherry-LacRnls constructs colocalizing with GFP-FIRRM in U2OS 2-6-5 cells and processed as in Fig.4D. Mean ± SD (*n* = 3). (**C**) Quantification of RAD51 foci intensity over mCherry signal at the *LacO* array foci. Mean ± SD (*n* = 3). (**D**) Representative images of U2OS 2-6-5 cells transfected with mCherry-LacR-FIGNL1 and GFP-FIRRM. Scale bar, 5 μm. (**E**) Quantification of U2OS 2-6-5 cells transfected with mCherry-LacRnls-FIRRM or -FIGNL1 and processed for RAD51 immunofluorescence. (**F**) Representative images of data shown in (E). Scale bar, 5 μm. (**G**) Schematic representation of FIRRM full length (FL) and truncated proteins (top). Quantification of GFP-tagged proteins colocalizing with mCherry-LacRnls-FIGNL1 in U2OS 2-6-5 cells is presented (bottom). Mean ± SD (*n* = 3). (**H**) Representative images of data quantified in (D). Scale bar, 5 μm. (**I**) WCE from U2OS cells depleted for FIGNL1 and transfected with mCherry-FIGNL1 and 3xFLAG-FIRRM FL or ∆WCF for 24 hours was processed for FLAG IP and analyzed by immunoblot. (**J**) WCE from U2OS cells overexpressing stable FIRRM FL or ∆WCF constructs depleted (black dot) for endogenous FIRRM and treated with Dox for 24 hours before analysis by immunoblot. * Denotes a nonspecific band. (**K**) Viability assay in WT, GFP-FIRRM WT, and GFP-FIRRM ∆WCF U2OS cells treated or not with siFIRRM before cisplatin treatment. Mean ± SD (*n* = 3). (**L**) Quantification of GFP-FIRRM WT and GFP-FIRRM ∆WCF U2OS cells with ≥5 RAD51 foci following treatment with cisplatin and Dox. Mean ± SD (*n* = 3). Data were analyzed with an unpaired *t* test with Welch’s correction (C, K, and L), a one-way Welch’s ANOVA test, and Dunnett’s multiple comparisons test (G). *P < 0.05, **P < 0.01, ***P < 0.001, and ****P < 0.0001.

FIGNL1 is a protein of 674 amino acids with three conserved domains: (i) a RAD51 binding domain [FIGNL1 RAD51 binding domain (FRBD)], (ii) an AAA^+^ ATPase domain, and (iii) a C-terminal Vps4 domain (Fig. 5A) (*9*, *17*). FIGNL1 has been shown to promote RAD51 dissociation from ssDNA (*9*, *17*). To define the role of the interplay between FIRRM and FIGNL1 during ICL repair, we first took advantage of our ability to detect the interaction of GFP-FIRRM with mCherry-LacRnls-FIGNL1 in the *LacO*/LacR assay (Fig. 4, C and D) to rapidly screen a panel of FIGNL1 truncation and mutant proteins (Fig. 5A). First, mCherry-LacRnls-FIGNL1 constructs were designed to remove or abolish the function of the previously described RAD51 binding domain (FRBD) (*17*) and AAA^+^ ATPase activity of FIGNL1 (*9*) (Fig. 5A and fig. S5A). In this experiment, FIGNL1 constructs lacking the ability to interact with RAD51 (∆FRBD or F295E mutant) still efficiently recruited GFP-FIRRM to the *LacO* array (Fig. 5B and fig. S5, B to D). Similarly, none of the single (K447A and D500A) and double (KDm: K447A/D500A) mutations of residues that are highly conserved within the AAA^+^ ATPase domain affected the interaction of FIGNL1 with FIRRM (Fig. 5B and fig. S5, B to D). The fact that FIGNL1 constructs encompassing amino acids 1 to 120 or 121 to 674 are unable to recruit FIRRM to the array suggests that the FIRRM binding motif of FIGNL1 spans over the intersection of these constructs (Fig. 5B). Consistently, the FIGNL1 fragments 1 to 360 efficiently promoted the accumulation of FIRRM in our assay.

As mCherry-LacRnls-FIGNL1 efficiently recruits RAD51 to the *LacO* array, we investigated whether this function can be regulated by the levels of FIRRM in cells. We observed a significant increase of RAD51 intensity at the mCherry locus in FIRRM-depleted U2OS 2-6-5 cells (Fig. 5C), suggesting that FIRRM negatively controls or counteracts FIGNL1-dependent RAD51 dynamics. Consistent with this hypothesis, ectopic expression of GFP-FIRRM had the opposite effect. It resulted in a reduced signal of RAD51 at the *LacO* array when compared to the expression of GFP alone (Fig. 5, C and D). While mCherry-LacRnls-FIGNL1 efficiently recruits RAD51 to the array, we observed that mCherry-LacRnls-FIRRM is unable to do the same (Fig. 5, E and F), suggesting that FIRRM does not directly interact with RAD51.

FIRRM is a protein of 853 amino acids containing a domain of unknown function (DUF4487) (Fig. 5G) (*19*). FIRRM also harbors a conserved WCF tripeptide motif essential for the interaction with FIGNL1 as described in the *LacO*/LacR assay (Fig.5, G and H, and fig. S5E) (*21*). Under these conditions, the C-terminal region of FIRRM is dispensable for the interaction with FIGNL1. Using Flag-tagged constructs of both WT and ΔWCF FIRRM, we confirmed the importance of this motif for FIGNL1 interaction (Fig. 5I). In contrast to FIRRM WT, reexpression of a GFP-tagged version of FIRRM ΔWCF mutant in FIRRM-depleted U2OS cells failed to restore FIGNL1 protein levels (Fig. 5J), confirming that FIRRM-FIGNL1 interaction is critical for the stability of this complex. Notably, reexpression of FIRRM ΔWCF restored the survival of cells to cisplatin (Fig. 5K and fig. S5F), prevented the formation of MN (fig. S5G), and facilitated RAD51 foci resolution in U2OS cells (Fig. 5L and fig. S5, H to J), suggesting that, once stabilized, FIRRM is capable of promoting RAD51 disassembly in a FIGNL1-independent manner.

### FIRRM binds ssDNA where it facilitates the unloading of RAD51 in vitro

**Fig. 6.**
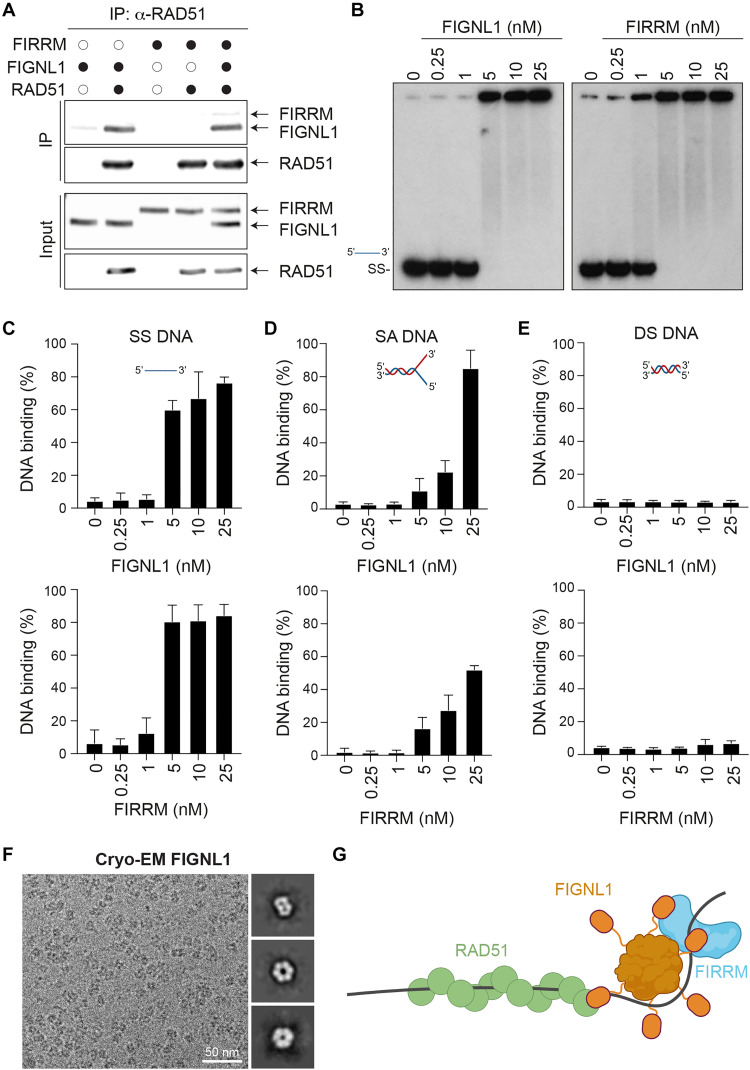
FIRRM interacts with ssDNA and SA DNA and forms a complex with FIGNL1 and RAD51. (**A**) Recombinant RAD51, FIGNL1, and FIRRM were processed for immunoprecipitation with an anti-RAD51 and analyzed by immunoblot with the indicated antibody. (**B**) Electrophoretic mobility shift assays (EMSA) of FIGNL1 and FIRRM on ssDNA. (**C**) Quantification of the percentage of DNA binding shown in (B). (**D** and **E**) Quantification of the percentage of DNA binding observed in EMSA experiment (fig. S6, C and D) on splayed arms and double-strand DNA substrates. (**F**) Representative cryo-EM micrograph of FIGNL1 bound to AMPPNP (1 mM) and MgCl_2_ (2 mM). Two D classes were obtained from 1287 particles. (**G**) Model of the RAD51-FIGNL1-FIRRM interaction in the presence of DNA.

To better understand how FIRRM may regulate RAD51 dynamics, we further investigated the biochemical activities and interactions with the RAD51 recombinase using full-length FIGNL1 and FIRRM either purified from bacteria or using Sf9 insect cells (iFIRRM and iFIGNL1) (fig. S6A). As reported previously (*17*), a strong interaction between FIGNL1 and RAD51 was detected following immunoprecipitation of RAD51 (Fig. 6A). In line with our previous observation in the *LacO*/LacR assay (Fig. 5, E and F), no direct interaction was detected between recombinant RAD51 and FIRRM in pull-down assays (Fig. 6A). A co-complex between RAD51 and FIRRM was detected upon adding FIGNL1 to the reaction likely reflecting the formation of the FIRRM-FIGNL1 complex with RAD51 in vitro.

During HR, the RAD51 recombinase nucleates on ssDNA to form an active nucleoprotein filament, which is highly regulated by several cofactors (*43*). To assess whether FIGNL1 and FIRRM bind ssDNA, we performed DNA binding assays. Both full-length FIGNL1 and FIRRM purified from bacteria and insect cells bind ssDNA in electrophoretic mobility shift assay (EMSA) in the presence or absence of a cross-linking agent (Fig. 6B and fig. S6B). In this type of assay, bacterially purified FIGNL1 and FIRRM proteins bind to a 60-nucleotide-long ssDNA probe and, to a lesser extent, a splayed arms DNA, but not to a double-strand DNA probe of the same size (Fig. 6, C to E, and fig. S6, B to D). During the purification of FIGNL1, we noticed that the protein purified form insect cells eluted from gel filtration column at a higher molecular weight than the predicted monomeric form (fig. S6E), suggesting that the protein forms multimers like other AAA^+^ ATPases. Cryo–electron microscopy (cryo-EM) and modeling approaches revealed that the protein preferentially associated into hexamers (Fig. 6F and fig. S6, E and F), with a central channel that is not large enough to accommodate ssDNA. On the basis of these observations, we propose a model where the functions of FIGNL1 and FIRRM at ICL repair rely on their ability to interact with DNA (Fig. 6G) (*44*, *45*). In this model, only FIGNL1 interacts with RAD51.

## DISCUSSION

### FIRRM contributes to HR-mediated ICL repair and beyond

While the initial steps involved in ICL repair have been extensively described, it remains largely unclear how ICL intermediates are processed and subsequently resolved. CRISPR-based functional genomics have been a powerful strategy to gain insight into several DNA repair pathways (*38*), including the recent characterization of SCAI as a modulator of repair pathway choice during ICL processing (*7*, *8*). Here, we delineated the contribution of FIRRM in the latter stage of ICL repair using a similar strategy. FIRRM has been recently proposed to play a significant role during the DNA damage response (*21*), and we showed here that lack of FIRRM impairs cell survival in response to a series of ICL-inducing agents. Under these conditions, FIRRM acts downstream of the FA pathway where it is functionally epistatic with the AAA^+^ ATPase FIGNL1 to resolve HR-mediated intermediates. Specifically, our findings revealed that FIRRM is required for the resolution of RAD51 from ssDNA, a phenotype that is reminiscent of the activity described for FIGNL1 (*9*). Consistent with a coordinate role of FIRRM and FIGNL1 in regulating HR-mediated DNA repair, a mutation in *figl1-1*, the ortholog of FIGNL1 in *A. thaliana*, is epistatic with *flip-1* mutation, the ortholog of FIRRM, in limiting meiotic crossover formation (*19*). Similar findings were observed in *O. sativa*, where targeting of MEICA, the ortholog of FIRRM in rice, is critical to prevent aberrant meiotic recombination and regulate crossover formation (*20*). More recently, FIRRM was found to genetically interact with factors implicated in the resolution of HR intermediates and the promotion of sister chromatid exchange in mammalian cells (*46*). While we could not detect the recruitment of endogenous FIRRM at ICLs, the endogenous tag version of FIRRM was recently shown to accumulate at MMC-induced damage and at laser UV-A micro-irradiation sites after psoralen treatment (*46*). FIRRM has also been detected at stalled replication fork by isolation of proteins on nascent DNA (iPOND) (*47*–*49*). This observation fits well with our findings that FIRRM regulates the recruitment of HR factors at stalled replication forks in the LacR assay and suggests that FIRRM plays a role beyond the repair of ICL-induced DSBs. The fact that FIRRM limits the formation of MN and the accumulation of 53BP1 NBs and prevents cellular senescence supports a model where FIRRM participates in the response to replication stress.

### FIRRM forms a complex with FIGNL1 to regulate RAD51 accumulation at DSBs

Previous reports identified FIGNL1 as a key regulator of the resolution of RAD51 foci in different biological contexts (*9*, *17*, *19*). Our systematic proteomics analysis of FIRRM identified FIGNL1 as a constitutive proximal interactor that we validated in cellula and by coimmunoprecipitation. Our data clearly show that the FIRRM-FIGNL1 interaction is necessary for the stability of the complex, reminiscent of what has been previously observed with the BRCA1/BARD1 heterodimer, where each member of the heterodimer controls the abundance, stability, and function of the other (*50*, *51*).

Our in vitro biochemical analysis provided better insight into the role of FIGNL1 and FIRRM during RAD51 dynamics. First, our characterization showed that FIGNL1, unlike FIRRM, strongly interacts with RAD51. Second, we noticed that both FIGNL1 and FIRRM preferentially interact with ssDNA, which is produced during the DNA resection step of HR. The integration of these data, along with previous findings demonstrating the ability of FIGNL1 to promote RAD51 filament disassembly in vitro (*9*), supports a model where FIRRM acts as a scaffolding platform for FIGNL1 hexameric ring structure to promote the release of ssDNA from RAD51 (Fig. 6G), thereby regulating RAD51 unloading at DSBs. While this model is attractive, it does not fully explain why FIRRM alone, when stabilized by the deletion of its WCF motif, promotes the resolution of RAD51. Additional biochemical investigation will be required to fully address this function. Similarly, further studies will be required to understand FIGNL1 and FIRRM cooperate with numerous other players, such as partner and localizer of BRCA2 (PALB2)/BRCA2 (*52*) and RPA1 related single stranded DNA binding protein, X-linked (RADX) (*53*, *54*), which respectively promote RAD51 nucleoprotein assembly and displace RAD51 from ssDNA, to regulate RAD51 filament dynamics in mammalian cells.

### FIRRM has independent function of FIGNL1 with potential relevance for cancer therapy

In this study, we found that the stability of FIRRM and FIGNL1 is interdependent. Whether both proteins are always in complex with one another remains however unknown. In species such as fungi and other model organisms such as *Caenorhabditis elegans* and *Drosophila melanogaster* (*19*), only FIGNL1 is conserved raising the possibility that it has conserved functions that are independent of FIRRM. Consistently, the predicted FIRRM interacting region is located in a less conserved region of FIGNL1. Depending on the cellular context, it is also possible that FIRRM forms different molecular complexes to preserve genomic stability, similar to other scaffolding proteins such as REV7 (MAD2L2: mitotic arrest deficient 2 like 2) [reviewed in (*55*)]. In line with this hypothesis, our structure-function analysis identified a mutant of FIRRM that stabilizes the protein in the absence of FIGNL1 and is functionally capable of resolving RAD51 foci, preventing MN formation and promoting survival to cisplatin. These findings suggest that FIRRM has functions that are independent of FIGNL1 in the regulation of RAD51 dynamics and ICL repair. Furthermore, our proteomic analysis identified ERCC6L as one of the top constitutive interactors of FIRRM, which may likely reflect a role during mitotic progression (*18*). These previously unidentified functions of FIRRM may be relevant in cancers where high levels of FIRRM is associated with poor outcome, including gliomas (*56*), and therefore may be a potential prognostic marker in multiple tumor types (*57*). Whether FIGNL1 is also expressed in these cancers is unknown. Together, our study identified FIRRM as a regulator of ICL-induced genomic instability and concomitant survival by modulating RAD51 dynamics. In line with this finding, high expression of FIRRM in cancer cells may provide an evolutionary advantage to resist stringent cellular conditions such as replication stress.

## METHODS

### Cell culture and plasmids transfections

All cell lines were maintained at 37°C and 5% CO_2_. Namalwa and Raji BL cell lines (a gift of J. Pelletier, McGill University) were cultured in RPMI 1640 medium (Wisent) and supplemented with 20% fetal bovine serum (FBS; Sigma-Aldrich) and 1% penicillin-streptomycin (Wisent). RPE1-hTERT p53 WT and p53^−/−^ cells (a gift of D. Durocher, University of Toronto) and IMR90 cells [National Institute of General Medical Sciences (NIGMS) Human Genetic Cell Repository I90-10] were cultured in Dulbecco’s modified Eagle’s medium (DMEM) medium (Wisent) and supplemented with 10% FBS and 1% penicillin-streptomycin. U2OS (American Type Culture Collection, number HTB-96) and U2OS 2-6-5 cell lines [a gift from R. Greenberg (University of Pennsylvania, Philadelphia, PA) (*30*)] were cultured in McCoy’s medium (Life Technologies) and supplemented with 10% FBS. Hela DR-GFP cells, U2OS DR-GFP, and U2OS SA-GFP cells (gift of J. Stark, City of Hope) were cultured in DMEM and McCoy’s medium (Wisent), respectively. These media were supplemented with 10% FBS and 1% penicillin-streptomycin.

RPE1-hTERT Cas9 p53^−/−^ *FIRRM*^−/−^ cells were generated by transduction of the parental cell line with lentiviral vectors harboring FIRRM-sgRNAs targeting exons 5 and 13 (table S4). Cells were selected with puromycin for 4 days, and clones were isolated by single-cell sorting. Knockout cells were validated using immunoblotting. Stable Cas9-expressing BL cell lines (Namalwa and Raji Burkitt cells) were generated using the LentiCas9-Blast vector as previously described (*58*) and validated for Cas9 expression by immunoblotting analysis. Cell lines used for all mTurboID-MS experiments were generated in HEK293 Flp-In T-REx cells as previously described (*14*) and a pool of stable transfectants selected with hygromycin (200 μg/ml; Multicell) and blasticidin (5 μg/ml;InvivoGen). Expression of bait proteins were induced with tetracycline (1 μg/ml) for 24 hours. U2OS Turbo-HA-2xStrep FIRRM WT/∆WCF or GFP-FIRRM WT/∆WCF cells were generated by transduction of the parental cell line with pCW57.1-Turbo-HA-2xStrep or pCW57.1-GFP expressing lentiviral vectors. Cells were selected with puromycin (2 μg/ml), and clones were isolated using a limited dilution approach. Expression of the constructs was induced with the addition of doxycycline (0.1, 1.0, or 5 μg/ml) and validated by immunoblotting analysis.

Plasmid transfections were done using Lipofectamine 2000 (Invitrogen) and TransIT-LT1 (Mirus) transfection agents according to the manufacturer’s instructions. Lentiviruses were produced as previously described (*59*). All cell lines were validated using short tandem repeat markers and tested negative for mycoplasma contamination.

### RNA interference

The pLKO shRNA plasmids against FIRRM were obtained from the McGill Platform for Cellular Perturbation as part of the TRC/RNAi Consortium from the Broad Institute. Nontargeting shRNA control was purchased from Addgene. Single siRNA duplex sequences targeting FIRRM, FIGNL1, FANCA, a custom nontargeting sequence, and SMARTPool siRNAs targeting RAD51 or CtIP were purchased from Horizon Discovery. Unless stated otherwise, all siRNAs were transfected at a concentration of 25 nM in a forward transfection for 48 hours using RNAiMAX (Invitrogen) according to the manufacturer’s instructions. Knockdowns were confirmed by immunoblotting or reverse transcription quantitative polymerase chain reaction (RT-qPCR) analyses. All siRNA, shRNA, and RT-qPCR primers sequences are detailed in table S4.

### Chemicals and sources of DNA damage

In the FOK1 system, DSBs were induced at the *LacO* array by inducing the nuclear localization [4-hydroxytamoxifen (100 nM, #3412, Tocris)] and stabilization (Shield-1 ligand, 0.5 μM; CIP-S1-0001, CheminPharma) of the ER-mCherry-LacR-FOK1-DD nuclease for 4 hours before immunofluorescence sample preparation. Replication fork stall at the *LacO* array was achieved by transfecting the empty mCherry-LacR fusion protein 24 hours before fixation. DNA damages were induced by exposing cells to either ionizing irradiation (IR 1 or 10 Gy) with a CellRad (Precision X-Ray Inc.), cisplatin treatments (Tocris; 1 μM for 3 or 24 hours at 250 nM in time course experiment and 2 μM in survival assay), MAF (1.30 and 1.57 μM in Namalwa and Raji screens, respectively; Toronto Research Chemicals), HU (4 and 1 mM in survival assay; Sigma-Aldrich), formaldehyde (150 μM; Sigma-Aldrich), MMC (150 nM; Sigma-Aldrich), talazoparib (3 μM; BMN 673, Selleck Chemicals), or UV (10 J/m^2^). UV irradiations were carried on with a germicidal lamp (243 nm).

### Plasmids

DNA sequences of sgRNAs were cloned into a modified form of LentiGuide-puro with BFP or mCherry marker as described in (*22*, *60*). The cDNA for FIRRM (MHS1010-202739778) and FIGNL1 (MHS6278-202759761) were obtained from Horizon Discovery, and coding sequences with respective attB1/B2 adapters were PCR-amplified and subcloned into a pDONR221 vector using a BP clonase II reaction according to the manufacturer’s instructions (Invitrogen), to generate ENTRY vectors. ENTRY clones containing FIRRM constructs (amino acids 1 to 175 and 1 to 735) and FIGNL1 constructs (amino acids 1 to 120, 121 to 674, 1 to 360, 361 to 674, and 295 to 344) were also generated using this approach. FIRRM deletion (∆WCF), FIGNL1 mutations and depletion constructs (∆295-344, F295E, K447A, D500A, and K477A/D500A) were directly introduced in ENTRY vectors using the QuickChange approach. QuickChange Site-Directed Mutagenesis was conducted using primers listed in table S4 using the QuickChange (Agilent) or Q5-Site-directed mutagenesis kit (New England Biolabs) and following the manufacturer’s protocol. LR recombining reactions were then conducted to transfer ENTRY constructs into pDEST-mTurboID (a gift from A.-C. Gingras, University of Toronto), pDEST pCW57.1-TurboID-HA-2xStrep (*61*), pDEST pCW57.1 GFP (*59*), pDEST-mCherry-LacRnls (a gift from D. Durocher, University of Toronto), or pDEST-FRT-TO-eGFP-nls. To produce recombinant proteins, full-length codon-optimized human FIGNL1 (UniProt, entry Q6PIW4) and FIRRM (UniProt, entry Q9NSG2) were purchased from GenScript and subcloned into a pGex6p2–glutathione *S*-transferase (GST)–His plasmid or a modified pFastBac dual vector (pfbdm) to be expressed in *Escherichia coli* and baculovirus, respectively. FIRRM and FIGNL1 were subcloned into pfbdm using NEBuilder HiFi DNA Assembly (New England Biolabs). The modified pFBDM expression vector includes an N-terminal GFP tag removable with tobacco etch virus protease. All sequences were validated through Sanger sequencing. A list of plasmids used in this study is provided in table S6.

### CRISPR- based genome-wide screening

Namalwa and Raji genome-wide screens were executed as previously described (*14*). Briefly, 245 million Namalwa and Raji cells expressing Cas9 were transduced with TKO v1 sgRNA lentiviral library at a multiplicity of infection of 0.2, ensuring coverage of at least 500-fold for each individual sgRNA represented in the cell population. Two days later, the selection of fully edited transduced cells was achieved by adding puromycin to the media at a final concentration of 2 μg/ml for 7 days. Following a 2-day recovery in fresh media without selection, cells were split into two pools, in triplicate, at a cell density of 45 million cells (D_0_). The first pools were treated with MAF at its IC_25_ (1.30 and 1.57 μM in Namalwa and Raji screens, respectively) and the second pool with DMSO as a vehicle. Cells were then cultured for 14 doubling times with puromycin at a concentration of 1 μg/ml. Cells were counted and passaged every 3 days at a cell density of 0.37 million cells/ml to maintain fold coverage of 500 cells per sgRNA, until D14. At each time point, cell pellets were frozen for subsequent genomic DNA purification. Genomic DNA isolation was performed as described in (*14*). For NGS library preparation, sgRNAs were amplified from genomic DNA using two rounds of nested PCR with inner oligo and outer oligo annealing. The initial outer PCR was performed with TaKaRa ExTaq DNA Polymerase Hot-Start Version polymerase (Takara) using forward and reverse outer primers (table S4). PCR products were pooled, and ~ 2% of the input was amplified using Hot Start Q5 polymerase to add Illumina HiSeq adapter sequences (table S4). The resulting product from each pooled sample was further purified following separation on an 8% 0.5× Tris/borate/EDTA (TBE) polyacrylamide gel. The library NGS was quantified using qPCR and analyzed by deep sequencing on the HiSeq 2500 Illumina platform. Reads were trimmed of NGS adapter sequences using the Cutadapt tool. Reads were aligned to the sgRNA library index file using Bowtie2. BAM files were generated using samtools, and total read count tables were subsequently generated using the MAGeCK count command. DrugZ algorithm was used to identify gene knockouts which were depleted or enriched from D14 populations in comparison to D0 (*16*).

### Acquisition of immunoblotting images

Immunoblot images were either acquired from film exposure or with an Azure Biosystems c300 Imaging System instrument. Briefly, membranes were exposed to Azure Radiance ECL (VWR) for 1 min before acquisition on the Azure apparatus with the sensitivity parameter set to “Normal” (1108 × 834). Whole pictures were adjusted for luminosity and contrast with the Adobe Photoshop software.

### CRISPR-based competition growth assay

Growth competition assays were conducted as described in (*22*) and (*60*). Briefly, RPE1-hTERT Cas9 p53^−/−^ were transduced with lentiviral particles of Lenti-mCherry-sgRNA-LacZ or Lenti-BFP-sgRNA-GOI (*FIRRM/FIGNL1/FANCA/MRE11*). Twenty-four hours after transduction, cells were selected with puromycin (15 μg/ml) for 4 days. BFP and mCherry-labeled cells were mixed at a 1:1 ratio and seeded in a six-well plate. At the initial time point (T0), cells were treated with cisplatin at 6 μM (IC_25_) or vehicle (DMSO), and the levels of each fluorophore were measured via flow cytometry. Cells were maintained under these conditions and subcultured for 20 days. The ratio of BFP to mCherry fluorescent cell population was assessed via flow cytometry every 5 days.

### Apoptosis

RPE1-hTERT Cas9 p53^−/−^ *FIRRM*^−/−^ clones 1 and 2 were seeded and treated with an overnight treatment of 4 μM cisplatin. The next day, cells were washed with Dulbeccos’s phosphate-buffered saline (D-PBS), and fresh medium was added to the plate. Forty-eight hours after cisplatin treatment, cells were processed for annexin V (BioLegend) and PI staining, following the manufacturer’s instructions. Analyses of annexin V and PI signals were done on at least 30,000 events acquired on a BD Fortessa (Becton Dickinson). Data were analyzed using the FlowJo software as previously described (*62*).

### Senescence-associated β-gal assay

Senescence-associated β-gal (SA β-gal) assays were performed as previously described (*63*). Briefly, shRNA expressing REP1 hTERT p53WT or p53^−/−^ or IMR90 cells were fixed with 0.5% glutaraldehyde in PBS, washed, and kept at 4°C in PBS supplemented with 1 mM MgCl_2_ (pH 6). Cells were stained with X-Gal solution containing potassium ferricyanide in PBS supplemented with 1 mM MgCl_2_ (pH 6). Images were acquired, and the percentage of SA β-gal–positive cells was quantified.

### Neutral comet assay

Neutral comet assay was performed as previously described in (*14*, *64*, *65*) and following the manufacturer’s specifications (Trevigen). Briefly, siRNA-treated U2OS cells were treated or not with 250 nM cisplatin 24 hours after tranfection. The next day, cells were washed off twice with D-PBS before the addition of fresh media. Cells were then harvested at the indicated time point, resuspended at 1 × 10^5^cells/ml in D-PBS, combined with prewarmed (37°C) molten LMAgarose at a ratio of 1:10, and evenly spread in CometSlides (Trevigen, catalog no. 4250-200-03). Comet slides were immersed into neutral lysis buffer overnight at 4°C and processed for electrophoresis and SYBR Gold (Invitrogen, catalog no. S11494) staining to reveal the comets as previously described (*14*). Images were acquired with the EVOS FL Cell Imaging System microscope, and the tail moments were quantified using ImageJ with the OpenComet plug-in (*66*). For each condition, at least 150 cells were analyzed.

### Cell viability and cell growth assays

siRNA-treated U2OS cells were exposed to different DNA-damaging agents as indicated. Forty-eight hours after the addition of treatment, cells were harvested and counted using a hemocytometer. Turbo-HA-2xStrep-FIRRM WT/∆WCF and GFP-FIRRM FL/∆WCF cell lines were supplemented with doxycycline either 6 hours after siRNAs transfection or 24 hours after seeding, and cell numbers were counted at the indicated time point following induction.

### Immunofluorescence microscopy

U2OS and U2OS 2-6-5 cells were grown on glass coverslips and fixed with 2% (w/v) paraformaldehyde (PFA) in PBS for 20 min at room temperature or with 100% methanol for 20 min at −20°C (RAD51 time-course experiments). When indicated, PFA-fixed cells were permeabilized cells with 0.3% (v/v) Triton X-100 in PBS for 20 min at room temperature. Cells were then incubated with blocking buffer (10% goat serum, 0.5% NP-40, and 0.5% saponin in 1× PBS) for 30 min at room temperature and then incubated with the indicated primary antibodies (table S5) for 2 hours at room temperature. After three washes with PBS, cells were incubated for 1 hour at room temperature with the indicated secondary antibodies (table S5) and 4′,6-diamidino-2-phenylindole (DAPI; 0.4 μg/ml). Coverslips were mounted onto glass slides with ProLong Diamond Antifade Mounting agent (Invitrogen). Images were acquired using a Zeiss LSM900 laser-scanning microscope equipped with a 40× and 63× oil lens. In all micrographs, dashed lines indicate the nucleus outline, and insets represent a 10-fold magnification of the indicated fields. Each quantification was done on at least three biological replicates, and at least 100 cells or 50 foci were counted in each experiment. Unless stated otherwise, significance was assessed by performing an unpaired *t* test with Welch’s correction.

### BrdU ssDNA detection assay

BrdU ssDNA detection assay was done as previously described (*32*). Briefly, siRNA-treated U2OS cells were incubated with 1 μM cisplatin and 10 μM BrdU (catalog no. B23151, Thermo Fisher Scientific) for 24 hours. Cell were then sequentially subjected to a nuclear preextraction buffer [10 mM Pipes (pH 7.0), 100 mM NaCl, 300 mM sucrose, 3 mM MgCl_2_, 1 mM EGTA, and 0.5% Triton X-100] and a cytoskeleton stripping buffer [10 mM tris (pH 7.4), 10 mM NaCl, 3 mM MgCl_2_, 1% Tween-20, and 0.5% sodium deoxycholate]. Following three washes in PBS, cells were fixed with 2% (w/v) PFA for 20 min on ice and then with 100% methanol for 5 min at −20°C. Cells were further permeabilized with 0.5% Triton X-100 for 10 min on ice before blocking with 3% bovine serum albumin (BSA) in PBS on ice for 1 hour. Cells were incubated with an anti-BrdU antibody overnight at 4°C. After three washes in PBS, cells were incubated with an anti-mouse Alexa Fluor 488 for 1 hour at room temperature followed by DAPI (0.4 μg/ml) for 5 min at room temperature. Coverslips were mounted and imaged as described above.

### mTurboID sample preparation for MS

HEK293 Flp-In cells expressing mTurboID-tagged protein or mTurbo tag alone were seeded in 150 mm plates in technical duplicates. Induction of fusion protein and proximity biotinylation was conducted as previously described (*14*, *62*). Cells were harvested, washed, and processed for streptavidin pull-down as previously described (*14*, *62*). Beads were washed with radioimmunoprecipitation assay buffer and 50 mM ammonium bicarbonate (ABC; pH 8.0) before being resuspended in 100 μl of ABC. Trypsin digestion was performed on beads overnight at 37°C. The next day, supernatant-containing peptides were collected and pooled before lyophilization by vacuum centrifugation.

Analysis of MS data was conducted on an Orbitrap Fusion (Thermo Fisher Scientific) as previously described with minor modifications (*62*). Raw files were analyzed with the Comet, XTandem! and Mascot search engines using the human RefSeq database (version 20170518). To estimate the interaction statistics, we used SAINTexpress [(*67*) (version 3.6.1)] on proteins with iProphet protein probability ≥ 0.9 and unique peptides ≥ 2. Each bait was compared against its respective negative control, which comprised of pulldowns from human embryonic kidney cells expressing the empty vector in triple technical replicates. Interactions displaying an averaged probability (AvgP) ≥ 0.7 were considered as statistically significant (table S3). Unfiltered contaminants, such as keratin, BirA*, and β-gal, were discarded in every bioinformatics analyses. Using the ProHits-Viz online tool (prohits-viz.org), we generated a dot plot representing the relative AvgSpec of identified proteins for both baits.

### Immunoprecipitation

U2OS cells were transfected with the indicated siRNA duplexes for 24 hours and then cotransfected with the indicated mCherry-LacR and/or 3× FLAG expression vectors. After 24 hours, cells were lysed in high-salt (HS) buffer (300 mM NaCl, 50 mM tris-HCl, 1 mM EDTA, and 1% Triton X-100) complemented with 1× cOmplete, EDTA-free Protease Inhibitor Cocktail (Roche), 20 mM *N*-ethylmethylamine, 1 mM NaF, and 0.2 mM Na_3_VO_4_. Cleared cell lysates were immunoprecipitated using either 1 μg of the indicated antibody coupled to 40 μl of packed protein G Sepharose beads (catalog no. GE17-0618-01, Sigma-Aldrich) for 3 hours at 4°C. Beads were washed four times with HS buffer + 0.1% BSA and eluted in 2× Laemmli buffer for immunoblotting.

### Protein expression and purification

*E. coli* BL21RP were transformed with pGex6p2-GST-FIGNL1-His plasmid or pGex6p2-GST-FIRRM-His plasmids. Colonies were resuspended in 1 liter of LB with antibiotics and grown at 37°C until an optical density between 0.4 and 0.6. The expression was induced using 0.1 mM isopropyl-β-d-thiogalactopyranoside and incubated overnight at 16°C. Bacteria were harvested by centrifugation, and the pellet was frozen on dry ice. Cells were lysed in PBS300 (PBS containing 300 mM NaCl), 1 mM dithiothreitol (DTT) and protease inhibitors, 0.05% Triton X-100, followed by four times 30-s on/off cycles of sonication on ice. The cell lysate was incubated with 1 mM MgCl_2_ and benzonase (15 U/ml) at 4°C for 45 min followed by centrifugation at 35,000 rpm for 1 hour. The soluble cell lysate was incubated with GST-Sepharose beads for 90 min at 4°C with gentle rotation. The beads were washed twice with PBS300 followed by incubation with PBS300 containing 5 mM adenosine triphosphate and 15 mM MgCl_2_, for 45 min at 4°C. GST-Sepharose beads were washed twice with PBS300 and once with P5 buffer [50 mM NaHPO_4_ (pH 7.0), 500 mM NaCl, 10% glycerol, 0.05% Triton-X-100, and 5 mM imidazole]. The beads were incubated with PreScission protease (60 U/ml) for 5 hours at 4°C in P5 buffer. The supernatant was collected and incubated with 400 μl of Talon beads for 1 hour at 4°C on gentle rotation. The beads were washed two times with P5 followed by two times with P30 (P5 buffer with a final concentration of 30 mM imidazole). Proteins were eluted twice with 200 μl of P500 (P5 buffer containing 500 mM imidazole), dialyzed for 1 hour at 4°C against the storage buffer [20 mM tris-HCl (pH 7.4), 200 mM NaCl, 10% glycerol, and 1 mM DTT], and stored in aliquots at −80°C.

### DNA-binding assays (EMSA) with fixation

γ-^32^P-labeled DNA oligonucleotides (100 nM; ssDNA–JYM 925, dsDNA–JYM 925 + 945, SA–JYM 925 + 926, and 5′biotin–JYM 1086) [sequence of oligonucleotides (company) are indicated in table S4] were added to tris-acetate binding buffer [40 mM tris-acetate (pH 7.5), 20 mM NaCl, 2 mM CaCl_2,_ 0.02% Tween, and 1 mM DTT]. An increasing amount of purified FIGNL1 or FIRRM (0 to 25 nM) was added to the DNA-buffer mix and was incubated at 37°C for 15 min. The total reaction volume was 10 μl. After incubation, the protein-DNA complexes were fixed with 0.2% glutaraldehyde for 15 min at 37°C. The reactions were subjected to electrophoresis at 150 V for 2.5 hours at room temperature on an 8% TBE acrylamide gel, and γ-^32^P-labeled DNA was visualized by autoradiography.

### Immunoprecipitation with recombinant protein

Protein A/G Sepharose beads (Pierce) were blocked in immunoprecipitation buffer (IPB) buffer [20 mM KPO_4_ (pH 7.4), 175 mM KCl, 0.5 mM EDTA, 0.5% NP-40, 10% glycerol, and 1 mM DTT] with BSA (1 mg/ml) for 20 min at room temperature. Anti-RAD51 14B4 (Novus) was added for 20 min at room temperature followed by the addition of the purified proteins for 15 min. Immunoprecipitates were washed four times in 1 ml of IPB buffer and visualized by Western blotting using the indicated antibodies.

### Cryo-EM sample preparation and data collection

FIGNL1 samples (10 μM) in 1× PBS were incubated with 2 mM MgCl_2_ and 1 mM adenylyl-imidodiphosphate (AMP-PNP), immediately applied to graphene oxide lacey carbon grids (300 mesh), and vitrified using an FEI Vitrobot Mark IV with 100% humidity and 4°C settings. Samples were blotted for 1.5 to 2 s before being plunged into the liquid ethane. Initial screening and optimization of conditions were performed on an FEI Tecnai G2 F20 operated at 200 kV at facility for electron microscopy research (FEMR) (McGill University) using a Gatan 626 single tilt cryo-holder. Screening images were recorded in a Gatan Ultrascan 4000, 4000 by 4000 charge-coupled device camera. Images taken at a nominal magnification of ×62,000 were imported into CryoSPARC (v4.1.1) (*68*). Contrast transfer function (CTF) parameter estimation was performed using the Patch CTF estimation. Blob picker was used to pick particles out of 15 micrographs to generate two-dimensional averages. The FIGNL1 model was generated using the AlphaFold prediction for the FIGNL1 monomer and applying the hexameric symmetry seen in other AAA^+^ ATPases.
