# Supplementary material for: FIRRM cooperates with FIGNL1 to promote RAD51 disassembly during DNA repair
Source: Sci Adv. 2023 Aug 9;9(32):eadf4082. doi: 10.1126/sciadv.adf4082 (PMC10411901; doi:10.1126/sciadv.adf4082)
Supplement: Supplementary file 1 — Supplementary Materials Figs. S1 to S6 Legends for tables S1 to S3 Tables S4 to S6 [file sciadv.adf4082_sm.pdf]

Science Advances
AAAS

Supplementary Materials for

FIRRM cooperates with FIGNL1 to promote RAD51 disassembly during DNA repair

Edgar Pinedo-Carpio *et al.*

Corresponding author: Alexandre Orthwein, alexandre.orthwein@emory.edu;
Amélie Fradet-Turcotte, amelie.fradet-turcotte@crchudequebec.ulaval.ca

Sci. Adv. **9**, eadf4082 (2023)
DOI: 10.1126/sciadv.adf4082

The PDF file includes:

Supplementary Materials
Figs. S1 to S6
Legends for tables S1 to S3
Tables S4 to S6

Other Supplementary Material for this manuscript includes the following:

Tables S1 to S3

Supplementary Materials

Gene Set Enrichment Analysis (GSEA) and Network interaction analysis

GSEAs were conducted individually for each DrugZ-ranked list from the Namalwa and Raji screens using the GSEA v4.3.0 package (https://www.gsea-msigdb.org/gsea/index.jsp) and using the Gene Ontology biological process gene sets. For the integrated pathway enrichment analysis, an average of the NormZ values acquired individually for both screens using DrugZ was obtained, and a merged ranked list was generated. Pathway enrichment analysis on the integrated ranked list was conducted using the package Enrichr. Network interaction analysis was performed using Cytoscape v 3.9.1 and the GeneMANIA package.

Construction of heatmaps

All heatmaps were generated with the ComplexHeatmap R package*(74)* by performing hierarchical clustering on drugs NormZ-scores derived from published CRISPR screens and limited to preys identified by our miniturboID assays. In legends, dark blue indicates NormZ scores $\leq$ -3 while bright red indicates scores $\geq$ 3. Similarly, we created a heatmap out of our miniturboID results using the log2-transformed SAF (Spectral Abundance Factor) metric, a normalization method calculated by dividing average spectral counts of preys by their respective protein length in amino acids.

GFP-based DNA repair Assays

DNA repair by HR (DR-GFP) or SSA (SA-GFP) of I-SceI-generated DSB were measured as previously described in the main text. Briefly, HeLa DR-GFP, U2OS DR-GFP or U2OS SA-GFP cells were seeded in 6-well plates at a density of 100,000 cells/well and were transfected with 25 nM of siRNA. Twenty-four hours later, cells were transfected with pCBA-SceI plasmid using Lipofectamine 2000 (Invitrogen). Cells were harvested 48 hrs post-transfection, and the percentage of GFP-expressing cells was measured by flow cytometry. Analysis of GFP-positive signal was done on at least 30 000 events acquired on a BD Fortessa (Becton Dickinson). Data were analyzed using the FlowJo software and presented as previously described in the main text.

Sulforhodamine B (SRB) Assay

RPE1-hTERT cells were seeded in 96-well plates at a density of 1000/cells per well. Twenty-four hours later, cisplatin and mafosfamide were added in a two-fold serial dilution from 50 to 0.097 µM. Survival was assessed four days after treatment using the sulforhodamine B (SRB) colorimetric assay. Briefly, after drug treatment cells were fixed by with 10% trichloroacetic acid (TCA, Bioshop Canada) for 4°C for 1 hr under gentle agitation. Cells were washed four times and plates were left air-drying overnight at room temperature. Next, cells were stained with 0.057% SRB (Sigma Aldrich) and incubated at room temperature for 30 minutes. Plates were then rinsed four times using 1% acetic acid and were left air-drying overnight. Protein content was solubilized in a 10 mM Tris base solution (pH 10.5) for 30 minutes at room temperature. Measurement of optical density (OD) at 510 nm was conducted using a FLUOstar Optima microplate reader. Background correction was conducted using the measurement of control wells with media. Treatments were performed in triplicate, averaged, and normalized to untreated control. IC50 concentrations were obtained using the slope's equation for log(concentration of drug) vs normalized OD.

EdU Cell Cycle kinetics Assay

EdU (5-ethynyl-2'-deoxyuridine) Cell cycle kinetic assays were conducted using the Click-iTTM EdU Alexa Fluor-647 kit (Invitrogen, Cat# 10419) following the manufacturer's protocol. Briefly, siRNA treated U2OS cell were sub-cultured to 60% confluency. Prior to cell collection, cells were incubated for 90 min with 10 μM Click-IT EdU solution. Cells were harvested and immediately processed with the Click-iTTM EdU Alexa Fluor-647 kit. Cells were counterstained with DAPI and at least 30,000 events were acquired on a BD Fortessa (Becton Dickinson). Data were analyzed using the FlowJo software.

Purification of protein from insect cells

FIGNL1 and FIRRM were produced using Sf9 insect cells infected with recombinant 3.5% baculovirus. Cells were grown at 27 °C for 48 h in I-Max medium (Wisent BioProducts) and harvested by centrifugation. Cell pellets were resuspended in PBS buffer at pH 7.4 (Fisher bioreagents) supplemented with 400 mM NaCl and protease inhibitors (1 mM Benzamidine, 1 mM PMSF, 5 μg/ml Leupeptin, and 0.7 μg/ml Pepstatin A), and lysed by sonication. The lysates were clarified by centrifugation at 39,000 g at 4 °C for 45 min. The supernatants were loaded into GFP-nanobody beads, equilibrated with PBS buffer, and incubated for 2 hours at 4 °C with gentle rocking. Contaminant proteins were washed away with PBS buffer supplemented with 400 mM NaCl, followed by a second wash with PBS buffer. The tags were removed by incubating the protein-bound GFP-nanobody beads with TEV protease at 4 °C overnight. Tagless FIGNL1 and FIRRM were resolved from tagged species over a HisTrap HP (Cytiva) pre-equilibrated with PBS buffer. Purified FIGNL1 and FIRRM were then concentrated using an Amicon Millipore 6 ml 50,000 MWCO centrifugal concentrator, flash-frozen, and stored at −80 °C.

DNA-binding assays (EMSA) without fixation

DNA binding of FIGNL1 and FIRRM was assessed using single-stranded, duplex and splayed 60 base pairs long DNA substrates (Integrated DNA Technologies). The duplex DNA substrate was generated by mixing the FAM-labeled ssDNA with equimolar amounts of complementary, unlabeled oligonucleotide in nuclease-free water. The splayed DNA substrate was generated by mixing SA-labeled and SA-unlabeled oligonucleotides and where only 30 nucleotides were complementary to each other. Increasing concentrations of FIGNL1 or FIRRM (0-2 μM) were incubated with the FAM-labeled oligonucleotide (10 nM) in 10 mM Tris pH 7.6, 40 mM NaCl, 1.2 mM β-mercaptoethanol, 40 mg/ml BSA and 4% glycerol for 40 minutes at 4 °C. For FIGNL1, samples contained 2 mM MgCl2 and 1 mM AMPPNP. Reaction mixtures (10 μL) were then resolved on 8% Tris-Glycine native polyacrylamide gels. Gels were imaged using the Sapphire™ Biomolecular Imager (Azure Biosystems).

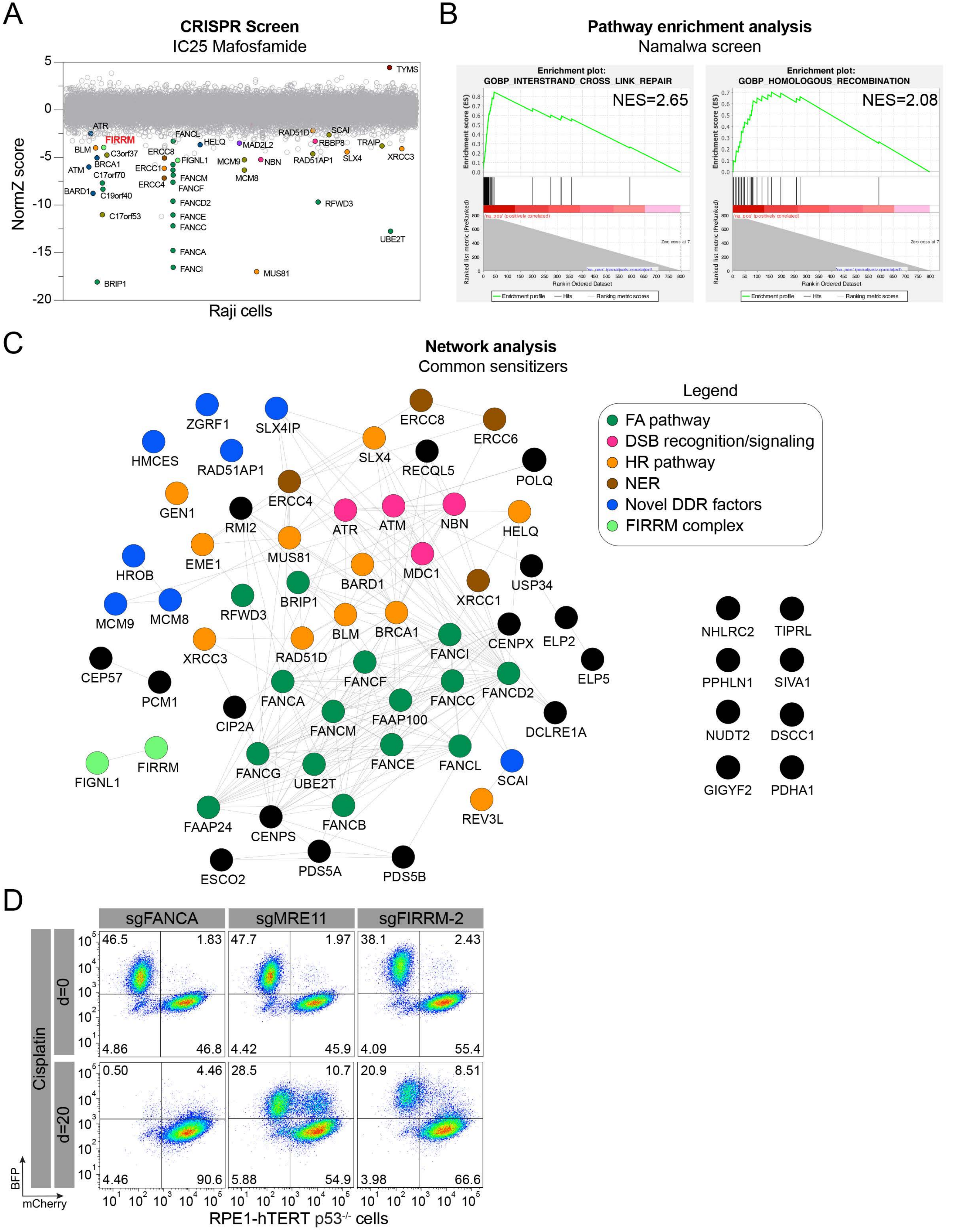

Pinedo Carpio, Dessapt *et al.* **Figure S1**

Fig. S1. Additional analysis of CRISPR screen performed in Raji and Namalwa cells, related to Fig. 1

(A) Horizontal scatter plot of DrugZ-generated ranking of the Raji mafosfamide CRISPR screen. NormZ values are plotted on the Y-axis, and gene names are plotted on the X-axis.
(B) Gene Set Enrichment Analysis (GSEA) analysis of the score obtained in the Namalwa screen for ICL repair and HR.
(C) Network analysis displaying protein physical interactions for sensitizer genes common to Namalwa and Raji CRISPR screens using Cytoscape and the GeneMANIA package.
(D) Representative scatter plots of BFP or mCherry-positive RPE1-hTERT p53-/- cells analyzed by flow cytometry and presented in Figure 1G.

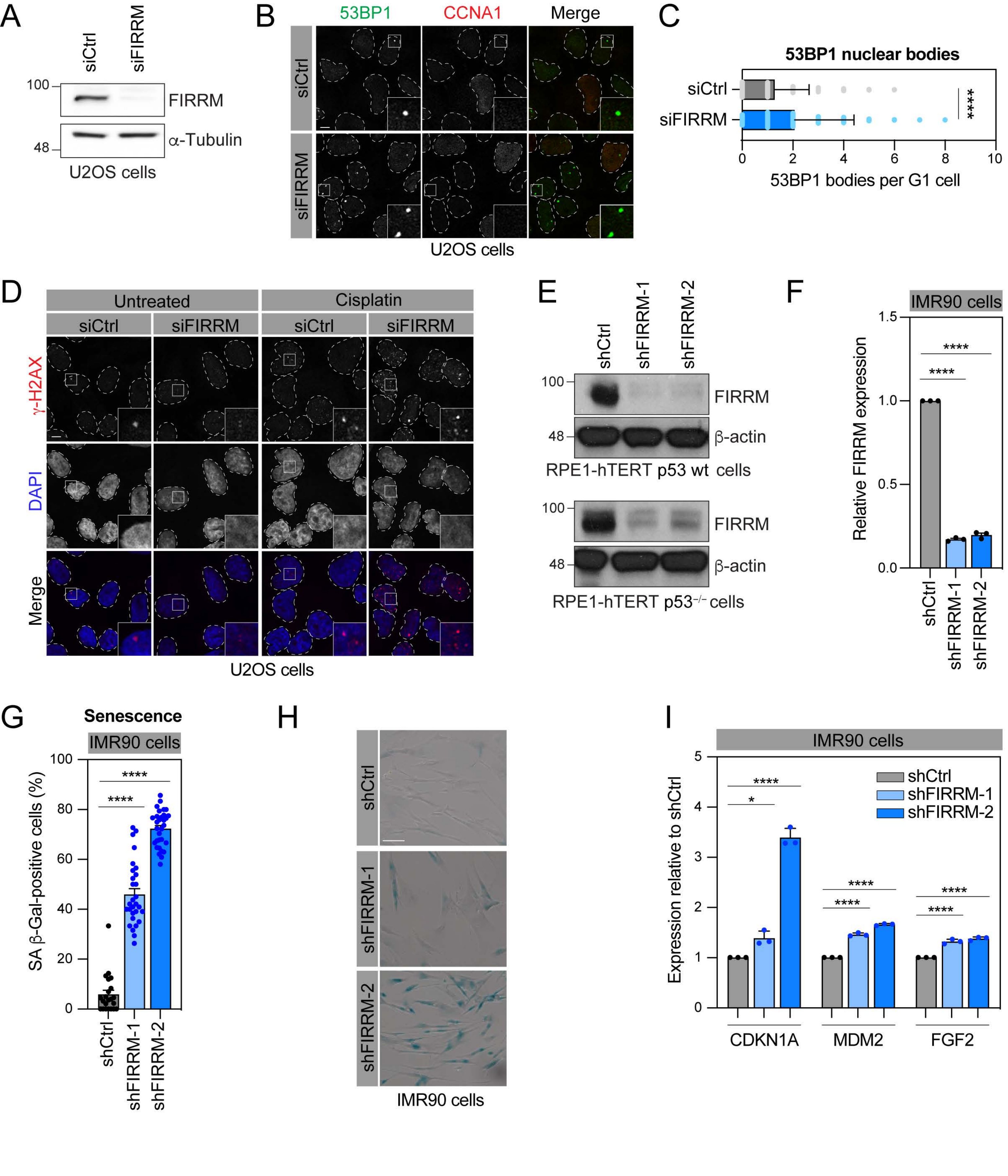

Pinedo Carpio, Dessapt *et al.* Figure S2

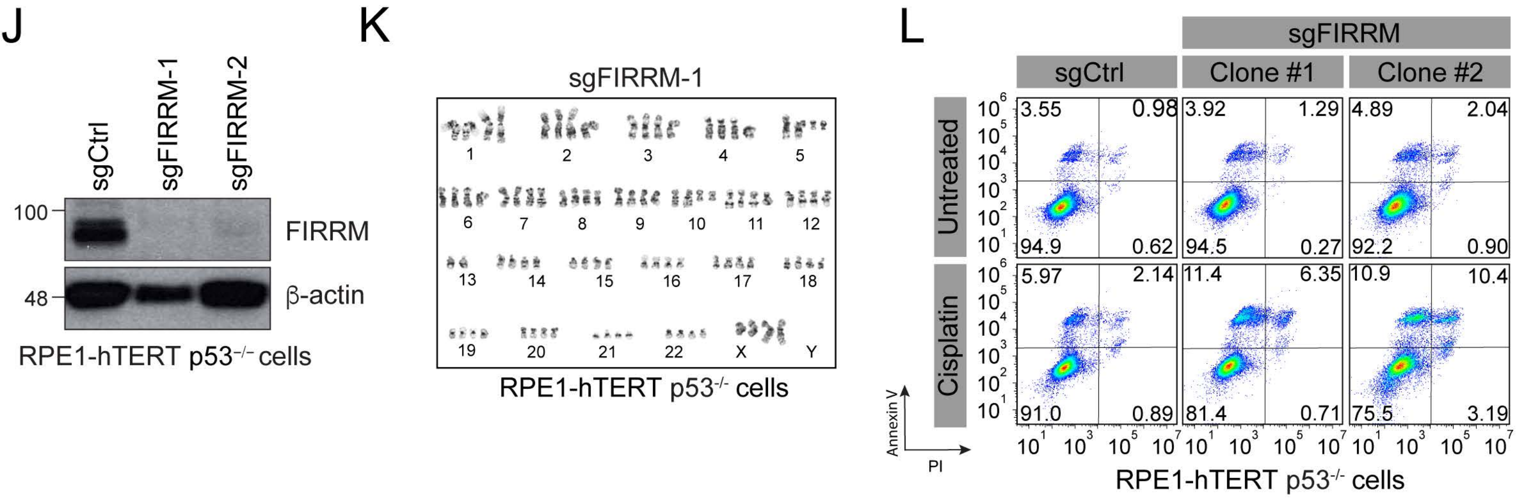

Pinedo Carpio, Dessapt *et al.* Figure S2

Fig. S2. Validation of FIRRM knockdowns and knockouts, analysis of 53BP1 nuclear bodies and additional senescence assays, related to Fig.2

(A) WCE of U2OS cells depleted or not for FIRRM were analyzed by immunoblot with an anti-FIRRM antibody. Anti-α-Tubulin was used as a loading control.

(B) Representative images of U2OS cells treated with a siFIRRM or non-targeting siRNA for 48 hrs. Cells were processed for 53BP1 and CCNA1 (Cyclin A) immunofluorescence. Scale bar = 5 μm.

(C) Quantification of 53BP1 nuclear bodies per CCNA1-negative cell as shown in (B). Data are represented as the mean ± SD (n = 3 independent experiments. A minimum of 100 cells were analyzed per condition per experiment).

(D) Representative images of data shown in Fig.2C.

(E) WCE of RPE1-hTERT WT or p53-/- cells depleted or not for FIRRM with the indicated shRNA were analyzed by immunoblot with an anti-FIRRM antibody. Anti-β-actin was used as a loading control.

(F) RT-qPCR for FIRRM was performed on IMR90 cells. Expression was normalized against GADPH and reported as relative to shCtrl. Data are represented as the mean ± SD (n = 3 independent experiments).

(G). Quantification of IMR90 cells depleted or not for FIRRM and stained with SA β-galactosidase. Data are represented as the mean ± SEM (n = 2 independent experiments with 3 technical replicates).

(H) Representative images of IMR90 cells depleted or not for FIRRM and stained with SA β-galactosidase. Scale bar = 100 μm

(I) RT-qPCR for CDKN1A (p21), MDM2 and FGF2 was performed on IMR90 cells. Expression was normalized against GAPDH and reported as relative to shCtrl. Data are represented as the mean ± SD (n = 3 independent experiments).

(J) WCE of RPE1-hTERT p53-/- clonal cells depleted or not for FIRRM with the indicated sgRNA were analyzed by immunoblot with an anti-FIRRM antibody. Anti-β-actin was used as a loading control.

(K) Karyotype representation of RPE1-hTERT p53-/- cells depleted (sgFIRRM-1) for FIRRM displaying aberrant chromosome copy numbers through metaphase analysis.

(L) Representative scatter plots of PI and/or Annexin V-positive RPE1-hTERT p53-/- cells analyzed by flow cytometry and presented in Fig.2I.

Data were analyzed with an unpaired t-test with Welch's correction (panel C) or an ordinary one-way ANOVA test with Dunnett's multiple comparison test (panels F, G and I). *$p<0.05$, ****$p<0.0001$

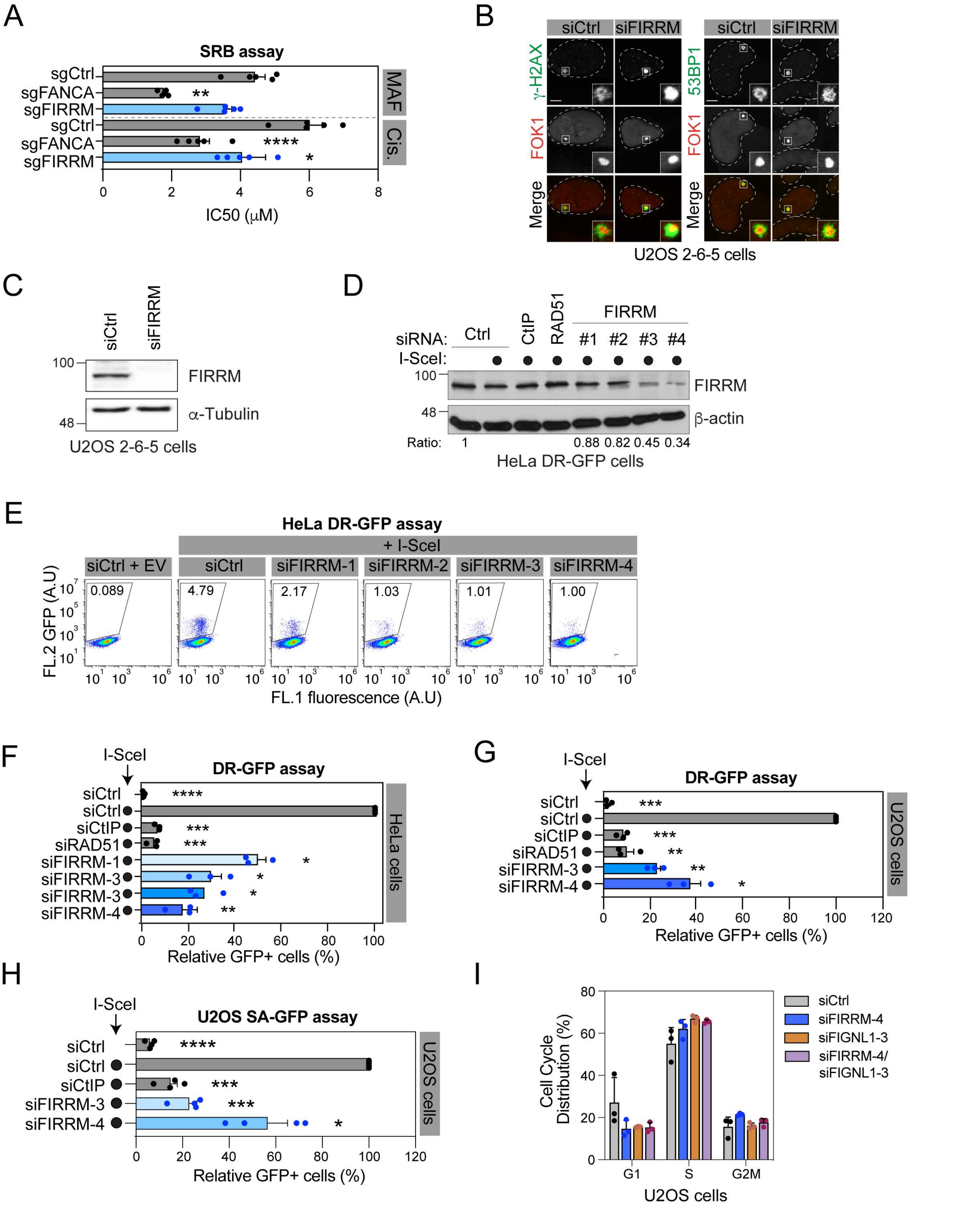

Pinedo Carpio, Dessapt *et al.* Figure S3

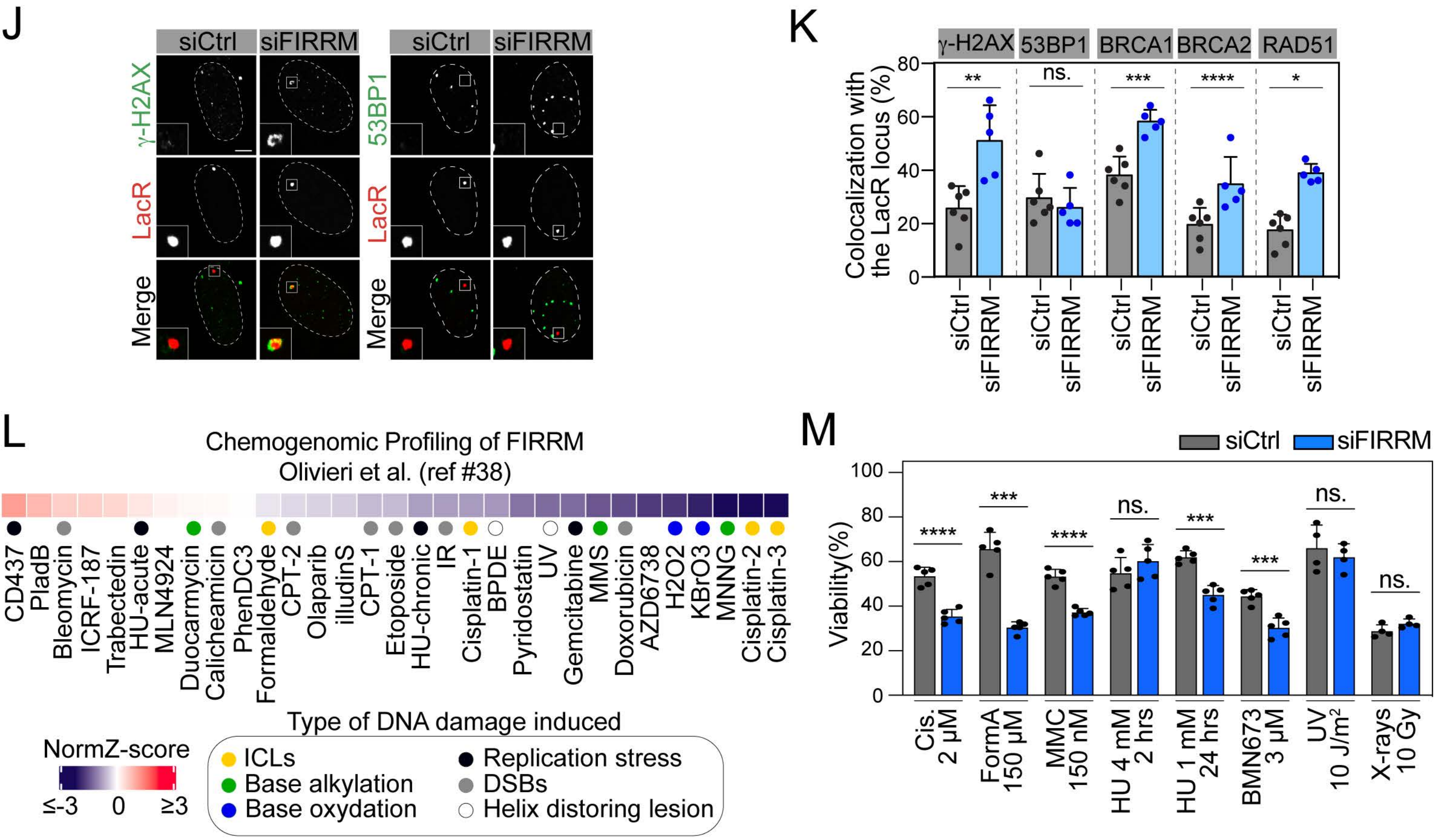

Pinedo Carpio, Dessapt *et al.* **Figure S3**

Fig. S3. Characterization of FIRRM knockdown and knockout cells related to Fig 3

(A) Quantification of RPE1-hTERT p53-/- cells survival in response to ICL-inducing agent. Cells were transduced with lentiviral particles containing sgRNAs against FIRRM, FANCA or a non-targeting control (sgCtrl). After selection with puromycin, cells were seeded in 96-well plates and treated with mafosfamide (MAF) or cisplatin (Cis.). Both drugs were added at a maximum concentration of 50 μM in a two-fold serial dilution until 0.097 μM. Data are represented as the mean ± SEM (n = 5 independent experiments).

(B) Representative images of U2OS 2-6-5 cells depleted or not for FIRRM. Cells were treated as in Figure 3E and processed for γ-H2AX (left) and 53BP1 (right) immunofluorescence. Scale bar = 5 μm.

(C) WCE of U2OS 2-6-5 cells depleted or not for FIRRM were analyzed by immunoblot with an anti-FIRRM antibody. Anti-α-Tubulin was used as a loading control.

(D) WCE of HeLa DR-GFP depleted or not for CtIP, RAD51 or FIRRM were analyzed by immunoblot with an anti-FIRRM antibody. Anti-β-actin was used as a loading control.

(E) Representative scatter plots of GFP-positive HeLa DR-GFP cells analyzed by flow cytometry and presented in (G). Flow cytometric profiles where green fluorescence (FL2) and auto orange fluorescence (FL1) are plotted on the y-axis and x-axis, respectively.

(F) Quantification of GFP-positive HeLa DR-GFP cells depleted or not for CtIP, RAD51 or FIRRM. Twenty-four hours after transfection of the indicated siRNA, cells were transfected with the I-SceI expression plasmid (●) or an empty vector. Data are represented as the mean ± SEM (n = 3 independent experiments, 30,000 cells quantified per experiment in each condition).

(G) Quantification of GFP-positive U2OS DR-GFP cells depleted or not for CtIP, RAD51 or FIRRM. Cells were processed and results analyzed as in Figure S3F.

(H) Quantification of GFP-positive U2OS SA-GFP cells depleted or not for CtIP or FIRRM. Twenty-four hours post siRNA transfection, cells were transfected with the I-SceI expression plasmid (●) or an empty vector. Data are represented as the mean ± SEM (n = 4 independent experiments, 30,000 cells quantified per experiment in each condition).

(I) Quantification of cell cycle distribution in U2OS cells depleted or not for FIRRM, FIGNL1 or both. Cells were fixed and processed for EdU analysis through flow cytometry. Data are represented as the mean ± SD (n = 3 independent experiments, at least 30,000 cells were analyzed per experiment in each condition).

(J) Representative images of U2OS 2-6-5 cells depleted or not for FIRRM. Cells were treated as in Figure 3K and processed for γ-H2AX (left) and 53BP1 (right) immunofluorescence. Scale bar = 5 μm.

(K) Quantification of the indicated protein with the empty mCherry-LacRnls construct. U2OS 2-6-5 cells were treated as in Figure 3K. Data are represented as the mean ± SD (n = at least 5 independent experiments, 50 foci quantified per experiment in each condition).

(L) Chemogenomic profiling using the NormZ-score from the CRISPR screens performed in RPE1 hTERT Cas9 p53-/- cells with the indicated DNA damaging agents as detailed in (38).

(M) Viability assay in cells treated with DNA damage-inducing agent. U2OS cells were treated with the indicated siRNA. Forty-eight hours later, cells were either exposed to 2 μM cisplatin (Cis.), 150 μM formaldehyde (FormA), 150 nM mitomycin C (MMC), 3 μM BMN-673 for 48 hrs, to 2 hrs of 4 mM hydroxyurea (HU) followed by 46 hrs recovery, to 24 hrs of 1 mM HU followed by 24 hrs recovery, to 10 J/m2 UV or 10 Gy X-rays followed by 48 hrs recovery. Cells were counted with a hemacytometer and normalized against the number of cells quantified in the untreated condition. Data are represented as the mean ± SD (n = 4 independent experiments).

Data were analyzed using a one-way Welch's ANOVA test with Dunnett's multiple comparison test (panels A, F, G and H) or with an unpaired t-test with Welch's correction (panels K and M). *$p<0.05$, **$p<0.01$, ***$p<0.001$, ****$p<0.0001$

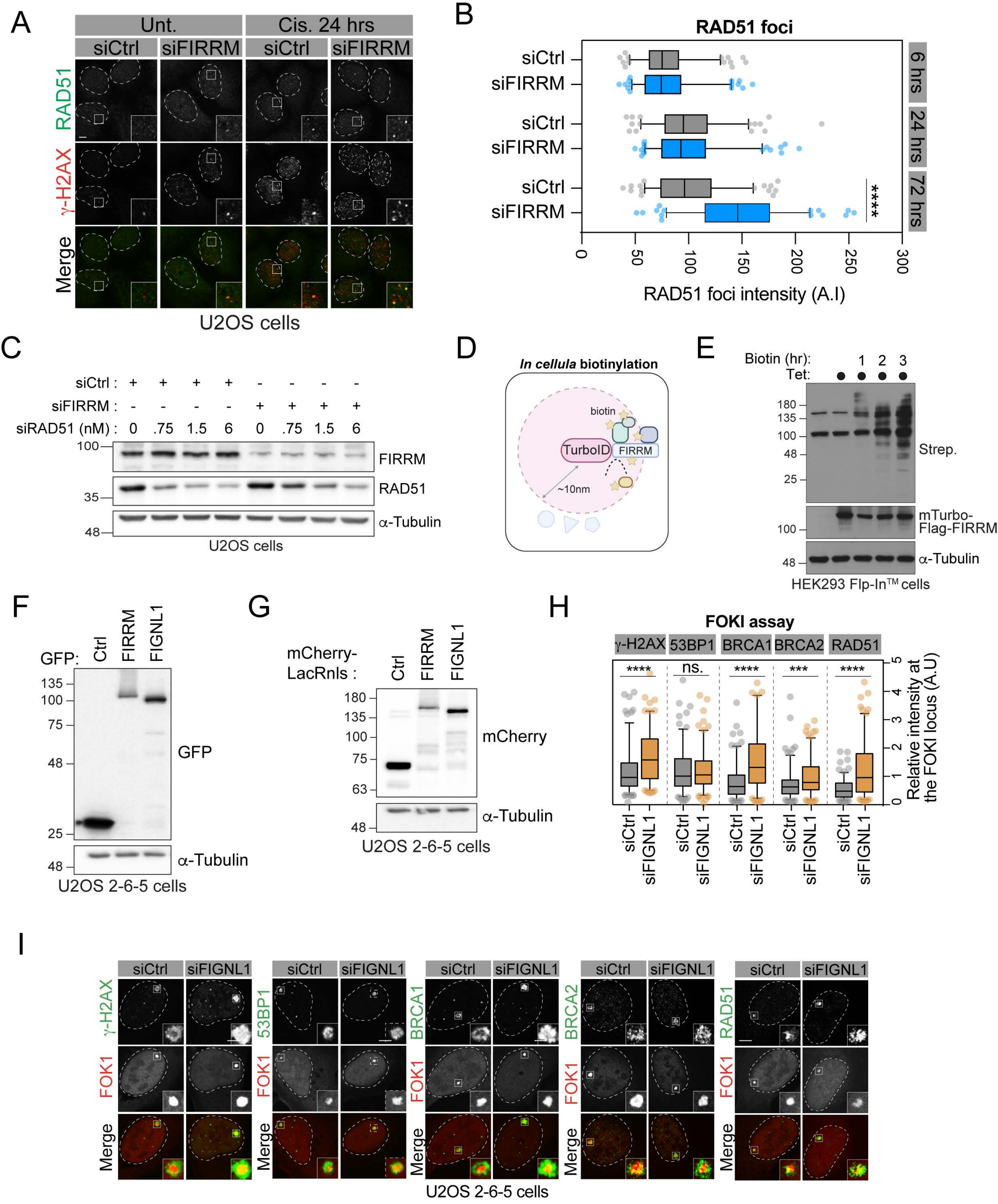

Pinedo Carpio, Dessapt *et al.* Figure S4

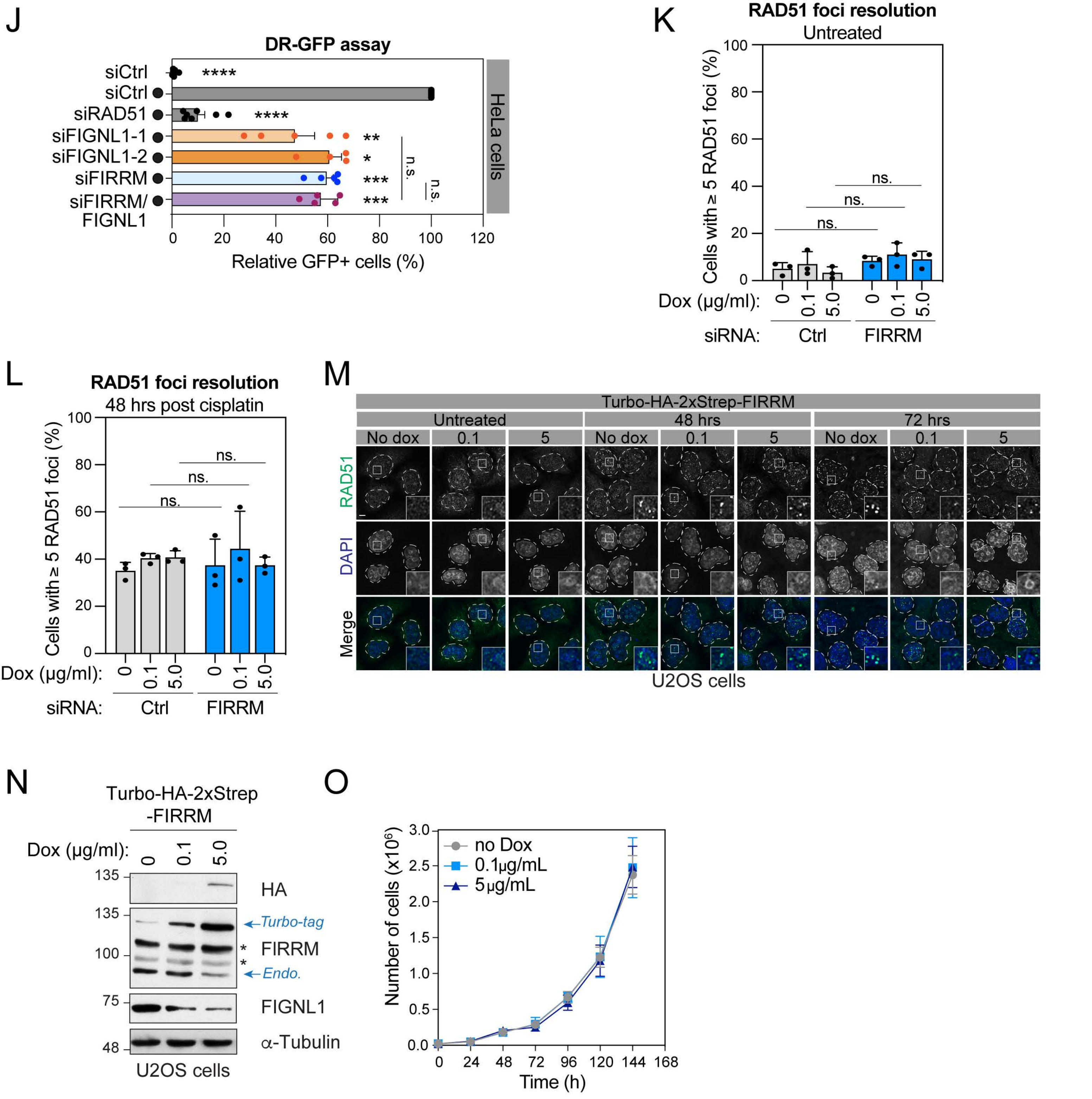
J
DR-GFP assay
siCtrl
siCtrl
siRAD51
siFIGNL1-1
siFIGNL1-2
siFIRRM
siFIRRM/
FIGNL1
****
****
**
*
***
***
n.s.
n.s.
HeLa cells
0 20 40 60 80 100 120
Relative GFP+ cells (%)
K
RAD51 foci resolution
Untreated
Cells with ≥ 5 RAD51 foci (%)
ns.
ns.
ns.
Dox (μg/ml): 0 0.1 5.0 0 0.1 5.0
siRNA: Ctrl FIRRM
L
RAD51 foci resolution
48 hrs post cisplatin
Cells with ≥ 5 RAD51 foci (%)
ns.
ns.
ns.
Dox (μg/ml): 0 0.1 5.0 0 0.1 5.0
siRNA: Ctrl FIRRM
M
Turbo-HA-2xStrep-FIRRM
Untreated
48 hrs
72 hrs
No dox 0.1 5 No dox 0.1 5 No dox 0.1 5
RAD51
DAPI
Merge
U2OS cells
N
Turbo-HA-2xStrep
-FIRRM
Dox (μg/ml): 0 0.1 5.0
135
135
100
75
48
HA
Turbo-tag
FIRRM
Endo.
FIGNL1
α-Tubulin
U2OS cells
O
no Dox
0.1μg/mL
5μg/mL
Number of cells (x10^6)
0 24 48 72 96 120 144 168
Time (h)

Fig. S4. Characterization of FIRRM in response to DNA DSBs, replicative stress, and other DNA damages, related to Fig 4.
(A) Representative images of data shown in Fig.4A.
(B) Quantification of RAD51 foci intensity from data shown in Fig. 4A.
(C) WCE of U2OS cells treated as in Fig.4C were analyzed by immunobloting with anti-FIRRM and anti-RAD51 antibodies. Anti-α-tubulin antibody was used as a loading control.
(D) Schematic representation of the biotinylation of FIRRM proximal endogenous proteins using TurboID technology. Cutoff for proximity tagging is reported at 10nm.
(E) WCE extracts of HEK293T Flp-InTM cells induced or not for mTurboID expression with 1μM tetracycline and supplemented or not for 50 μM biotin were analyzed by immunoblot with anti-streptavidin and anti-Flag antibodies. Anti-α-tubulin was used as a loading control.
(F-G) WCE extracts of U2OS 2-6-5 cells transfected with the indicated GFP- (F) or mCherry-LacRnls- (G) proteins were analyzed by immunoblot with anti-GFP or anti-mCherry antibodies. GFP alone was used as control (Ctrl). Anti-α-tubulin was used as a loading control.
(H) Quantification of the ratio of indicated protein foci intensity over ER-mCherry-LacR-FOK1-DD foci intensity as shown in (I). Data are represented as the mean ± SD (n = 4 independent experiments, 50 foci quantified per experiment in each condition).
(I) U2OS 2-6-5 cells were depleted or not for FIGNL1 (for 48 hrs and ER-mCherry-LacR-FOK1-DD expression was induced for 4 hrs before fixation. Cells were then processed for γ-H2AX, 53BP1, BRCA1, BRCA2 or RAD51 immunofluorescence. Scale bar = 5 μm.
(J) Quantification of GFP-positive HeLa DR-GFP cells depleted or not for RAD51, FIGNL1 and/or FIRRM. HeLa cells containing a DR-GFP reporter cassette were transfected and analyzed as described in Figure S3H. (●) represent I-SceI transfected cells. Data are represented as the mean ± SEM (n = at least 5 independent experiments, 30,000 cells quantified per experiment in each condition).
(K-L) Quantification of GFP-FIRRM WT and GFP-FIRRM ΔWCF U2OS cells with ≥ 5 RAD51 foci in non-treated cells (NT) (K) and 72 hrs following treatment with cisplatin (L). Mean ± SD (n = 3 independent experiments).
(M) Representative images of the data quantified in Fig. 4M and S4K-L (siFIRRM only). Cells were fixed and processed for RAD51 (green) and γ-H2AX (red) immunofluorescence. Scale bar = 5 μm.
(N) WCE of Turbo-HA-2xStrep-FIRRM U2OS cells treated with the indicated amount of Dox were analyzed by immunoblot with anti-FIRRM, anti-HA and anti-FIGNL1 antibodies. Anti-α-tubulin antibody was used as a loading control. * Denotes a non-specific band.
(O) Turbo-HA-2xStrep-FIRRM U2OS cells treated or not with the indicated amount of Dox were grown over 7 days and cell number was assessed every 24 hrs.
Data were analyzed with an unpaired t-test with Welch's correction (panels B, H, K and L) or using a one-way Welch's ANOVA test with Dunnett's multiple comparison test (panel J). *$p < 0.05$, **$p<0.01$, ***$p<0.001$, ****$p<0.0001$

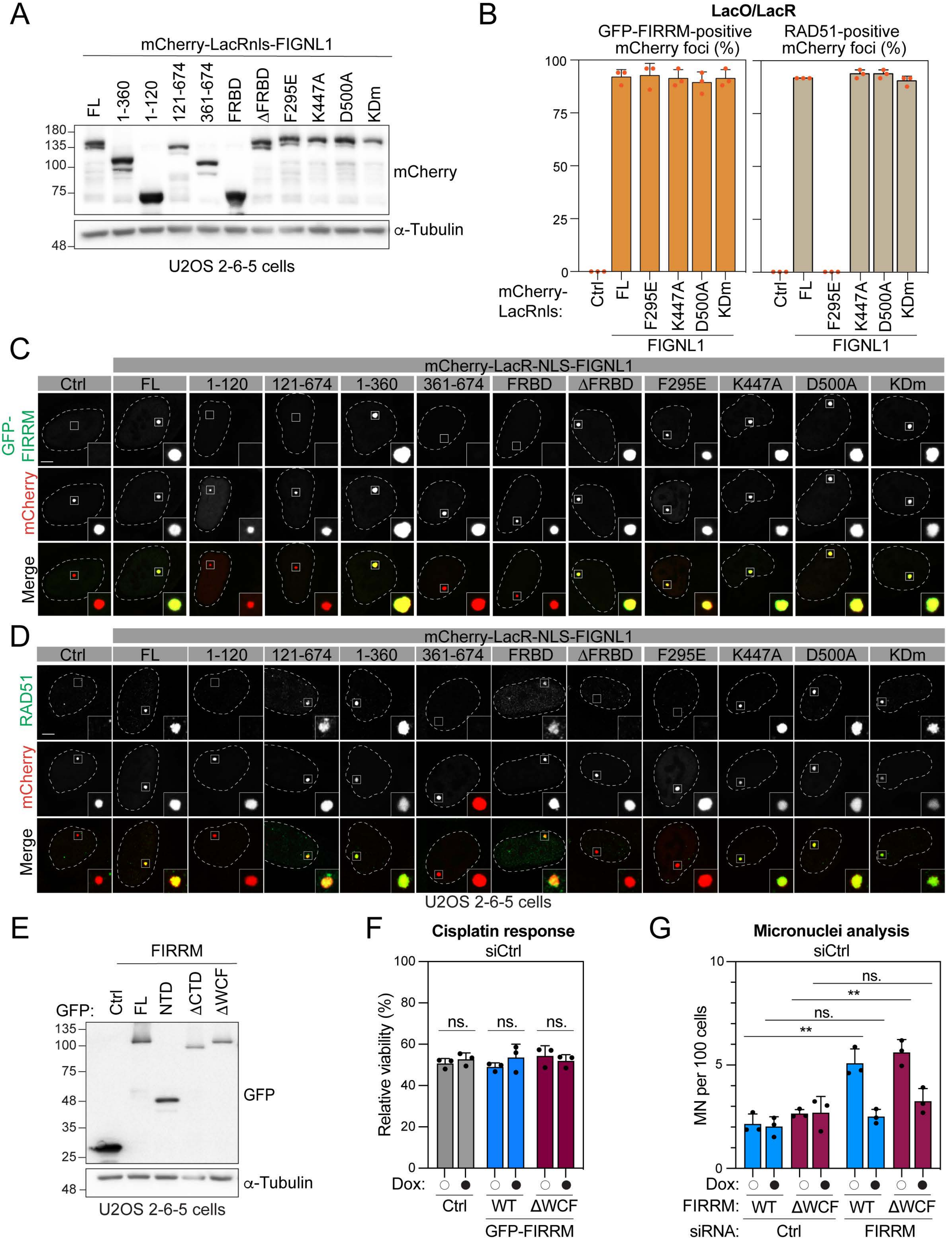

Pinedo Carpio, Dessapt *et al.* Figure S5

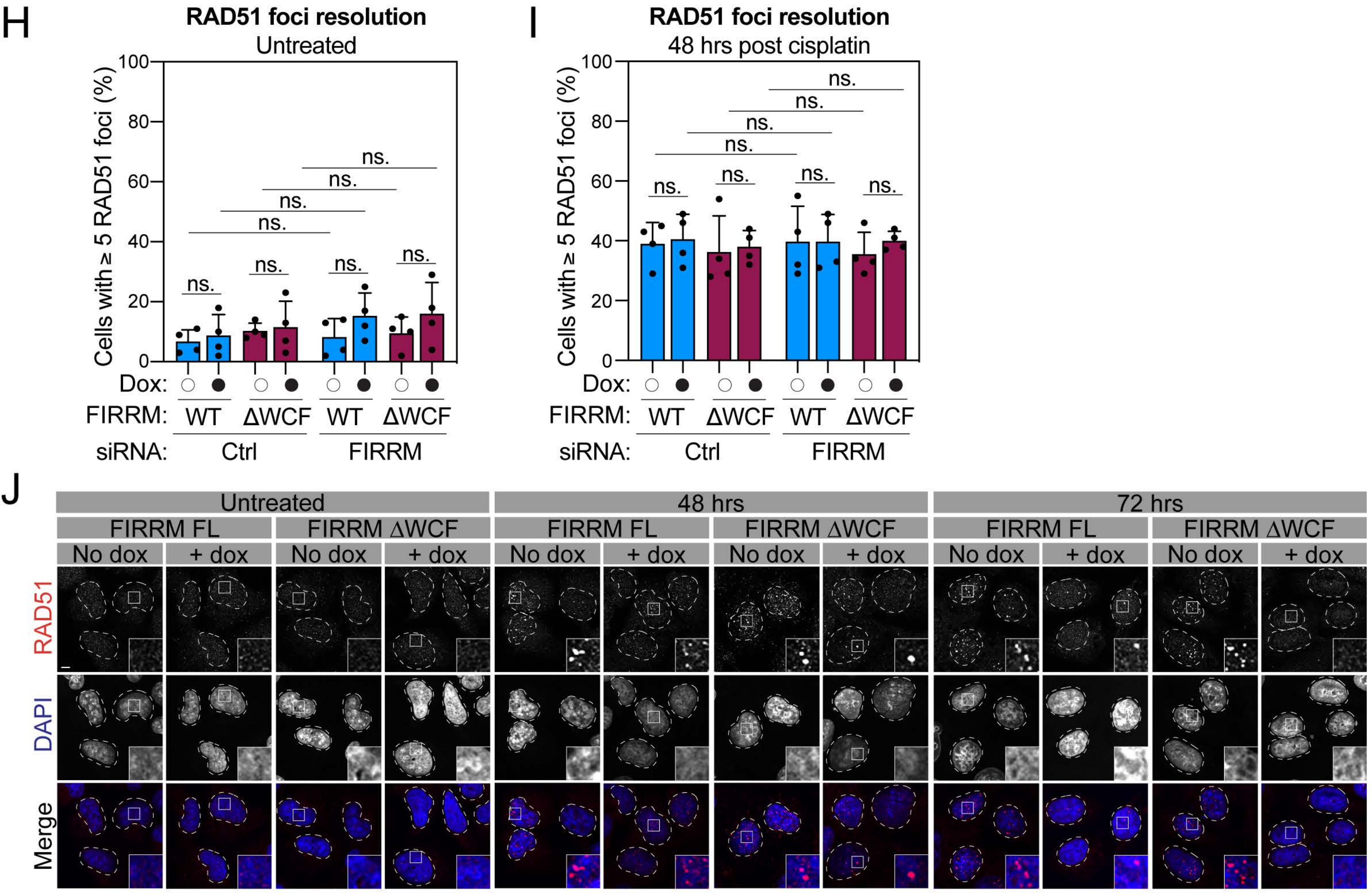

Pinedo Carpio, Dessapt *et al.* Figure S5

Fig. S5. Expression levels and cellular localization of mCherry-LacRnls-FIGNL1 and GFP-FIRRM constructs used in Fig. 5.

(A) WCE of U2OS 2-6-5 cells transfected with the indicated mCherry-LacRnls-FIGNL1 constructs were analyzed by immunoblot with an anti-mCherry antibody. Anti-α-tubulin antibody was used as a loading control. KDm: K447A / D500A double mutant.

(B) Quantification of mCherry-LacRnls constructs colocalizing with GFP-FIRRM (left) or RAD51 (right) in U2OS 2-6-5 cells. Cells were treated and processed as described in Figure 4C. Data are represented as the mean ± SD (n = 3 independent experiments, 50 cells quantified per experiment in each condition).

(C-D) Representative images of data from (B). U2OS 2-6-5 cells were transfected with indicated mCherry-LacR construct and then fixed, processed for RAD51 immunofluorescence (D), and mounted for confocal analyses. Scale bar = 5 µm.

(E) U2OS 2-6-5 cells were transfected with the indicated GFP-FIRRM constructs and analyzed by immunoblot with a GFP antibody. GFP was used as control. Anti-α-tubulin antibody was used as a loading control.

(F) Quantification of cell survival in response to cisplatin in GFP-FIRRM WT and GFP-FIRRM ΔWCF U2OS. Cells were treated with a Ctrl siRNA and supplemented or not with 1 µg/mL Dox prior to treatment with 1 µM cisplatin. Cells were then processed as in Figure 5K. Data are represented as the mean ± SD (n = 3 independent experiments)

(G) Quantification of the number of micronuclei in untreated GFP-FIRRM WT and GFP-FIRRM ΔWCF U2OS cells. Cells were treated with the indicated siRNA and fixed 48 hrs later. Data are represented as the mean ± SD (n = 3 independent experiments, at least 100 cells quantified per experiment in each condition).

(H-I) Quantification of GFP-FIRRM WT and GFP-FIRRM ΔWCF U2OS cells with ≥ 5 RAD51 foci in untreated cells (Unt.) (H) or 48 hrs following treatment with cisplatin (I). Mean ± SD (n = 3 independent experiments).

(J) Representative images of data shown in Figs.5L and S5H-I (siFIRRM only).

Data were analyzed with an unpaired t-test with Welch's correction (panels F, G, H and I). *$p < 0.05$, **$p<0.01$

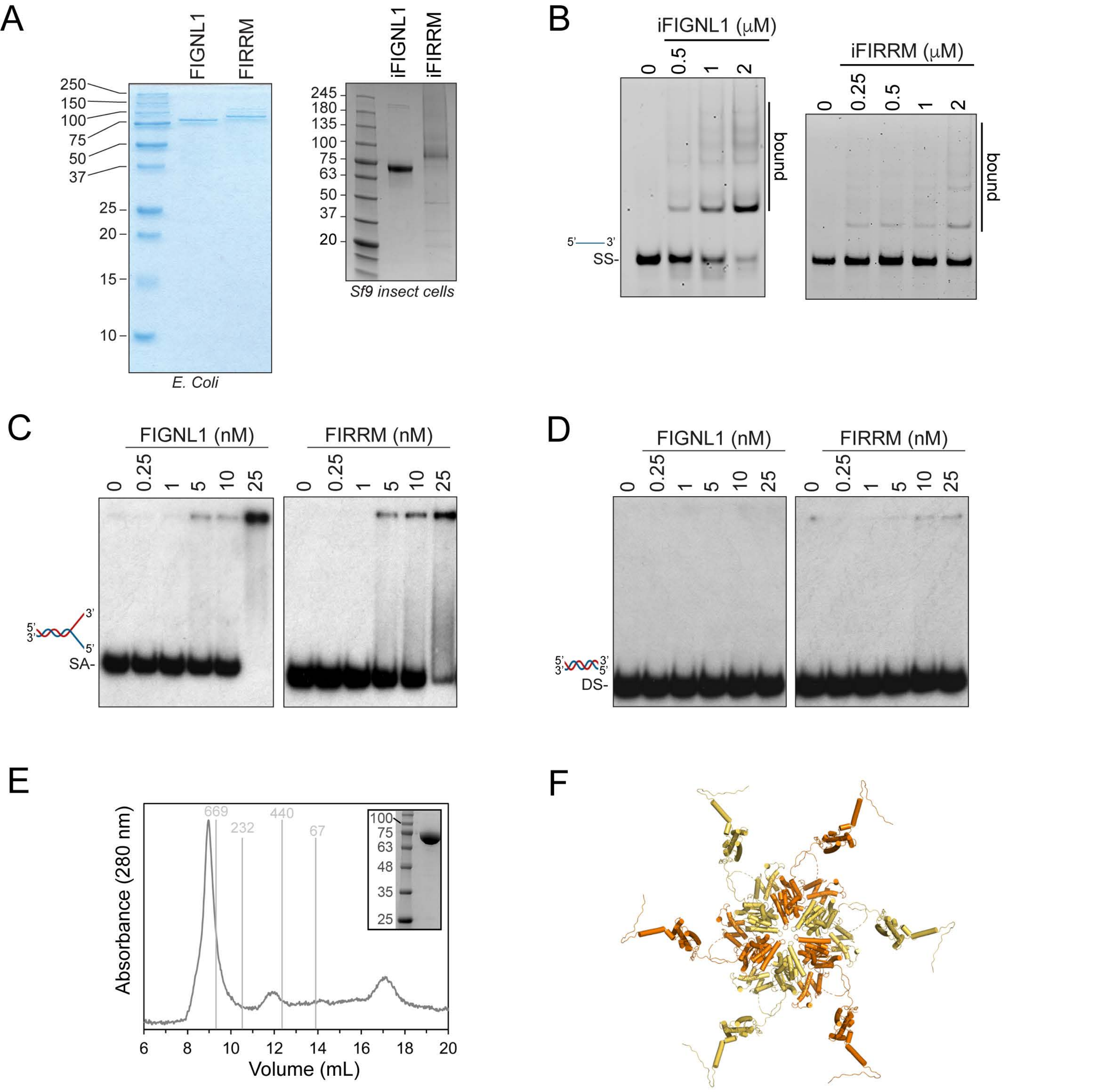

Pinedo Carpio, Dessapt *et al.* Figure S6

Fig. S6. FIRRM interacts with SS and SA DNA and forms a complex with FIGNL1 and RAD51.

(A) Coomassie stained SDS-PAGE of recombinant full-length FIGNL1 and FIRRM purified from bacteria or insect cells as indicated.

(B) EMSA of a 60-mer single-strand DNA with FIGNL1 (left) and FIRRM (right).

(C-D) Comparative electrophoretic mobility shift assays of splayed arm DNA (C) and double-stranded DNA (D) with FIGNL1 and FIRRM.

(E) iFIGNL1 elution profile over a Superdex 200 Increase 10/300 size exclusion chromatography column in the presence of ADP (1 mM) and MgCl2 (2 mM). The inset shows a Coomassie stained gel of the iFIGNL1 sample loaded into the column. Elution volumes for the molecular weight markers (thyroglobulin (669 kDa), ferritin (440 kDa), catalase (232 kDa), ovalbumin (67 kDa)) are indicated.

(F) Molecular model of FIGNL1 generated by imposing the canonical hexameric symmetry of AAA+ ATPases to the AlphaFold model of the FIGNL1 monomer

Table S1. NormZ values for Namalwa and Raji CRISPR screens. Provided as an Excel file.

Table S2. Genes Ontology analysis; related to Fig.1D. Provided as an Excel file.

Table S3. Hits from mTurboID of FIRRM; related to Fig.4A. Provided as an Excel file.

Table S4. Primers, sgRNAs, shRNAs and siRNAs sequences used in this study.

Name	Sequence 5'-3'	Used for[a]
sgCtrl LacZ	CCCGAATCTCTATCGTGCGG	KO in RPE1-hTERT Cas9 $p53^{-/-}$
sgFIRRM-1 Exon 13	TCCCTCACTGTTTGCTGAAC	KO in RPE1-hTERT Cas9 $p53^{-/-}$
sgFIRRM-2 Exon 5	TTATATGAAGGACTGAGGAG	KO in RPE1-hTERT Cas9 $p53^{-/-}$
sgFANCA	CCACAGCATGCATGTCGGGA	KO in RPE1-hTERT Cas9 $p53^{-/-}$
sgMRE11	GCAATCATGACGATCCCACA	KO in RPE1-hTERT Cas9 $p53^{-/-}$
sgFIGNL1-1	GAAGACCCTGATGCACGCTG	KO in RPE1-hTERT Cas9 $p53^{-/-}$
sgFIGNL1-2	TTCTAAATGGGTAGGTGAGG	KO in RPE1-hTERT Cas9 $p53^{-/-}$
shCtrl scramble Addgene #1864	CCTAAGGTTAAGTCGCCCTCG CTCGAGCGAGGGCGACTTAAC CTTAGG	KD in RPE1-hTERT WT and $p53^{-/-}$, and in IMR90
shFIRRM-1 TRCN0000163284	GCTTCCTGACTATGTTCGTTT	KD in RPE1-hTERT WT and $p53^{-/-}$, and in IMR90
shFIRRM-2 TRCN0000165342	GCAAGTTTCCTCCAAGCCTTT	KD in RPE1-hTERT WT and $p53^{-/-}$, and in IMR90
siFIRRM-1 D-020930-01	CAUAGUGGCUCAUCUGAUA	KD in U2OS, HeLa DR-GFP, U2OS DR-GFP, U2OS SA-GFP & RPE1-hTERT $p53^{-/-}$
siFIRRM-2 D-020930-02	AGACCUCGCUACUUAAAUU	KD in U2OS, U2OS DR-GFP, U2OS SA-GFP, HeLa DR-GFP & RPE1-hTERT $p53^{-/-}$
siFIRRM-3 D-020930-03	GAAACGACAACCAGGAUAU	KD in U2OS, U2OS DR-GFP, U2OS SA-GFP, HeLa DR-GFP & RPE1-hTERT $p53^{-/-}$

siFIRRM-4<br>D-020930-04	AGAGAUAGUUCCACAGUGU	KD in U2OS, U2OS 2-6-5, U2OS DR-GFP, U2OS SA-GFP, HeLa DR-GFP & RPE1-hTERT p53$^{-/-}$
siFIGNL1-1<br>D-019091-03	GAGCAUGAAUCUUCUAGAA	KD in HeLa DR-GFP
siFIGNL1-2<br>D-019091-17	GCACAGAUAUUACGCAUUC	KD in U2OS, U2OS DR-GFP & HeLa DR-GFP
siCtIP<br>M-011376-00	Smart pool<br>D-011376-01: GAGCAGACCUUUCUCAGUA<br>D-011376-02: GAAGUGAACAAGAUCAUUA<br>D-011376-03: CAACCAAGAUGUAUCCUUU<br>D-011376-04: GAAUAGGACUGAGUACGGU	KD in U2OS DR-GFP, HeLa DR-GFP & U2OS SA-GFP, BrdU assay
siRAD51<br>M-003530-04	Smart pool:<br>D-003530-02: GAAGCUAUGUUCGCCAUUA<br>D-003530-05: GCAGUGAUGUCCUGGAUAA<br>D-003530-07: CCAACGAUGUGAAGAAAUU<br>D-003530-08: AAGCUAUGUUCGCCAUUAA	KD in U2OS, U2OS 2-6-5, U2OS DR-GFP & HeLa DR-GFP
siFANCA custom	GGACAGAUCUGCACGGCUC	KD in U2OS
siCtrl-1<br>custom	UGGUUUACAUGUCGACUAA	KD in U2OS and U2OS 2-6-5
siCtrl-2<br>D-001210-03	UGGUUUACAUGUUUUCUGA	KD in U2OS and U2OS 2-6-5
GAPDH FW	CAACGTGTCAGTGGTGGACC	RT, qPCR
GAPDH RV	TCGTTGAGGGCAATGCCAGC	RT, qPCR
FIGNL1 FW	ACCAGCCGCAGGTGAAAAC	qPCR
FIGNL1 RV	ACAATGCTCCTTGATGCTGC	qPCR
TKO library outer-PCR primer FW	AGGGCCTATTTCCCATGATTCCTT	PCR
TKO library outer-PCR primer RV	TCAAAAAAGCACCGACTCGG	PCR
TKO library inner-PCR primer trueseq i5 1	AATGATACGGCGACCACCGAGATC<br>TACACTATAGCCTACACTCTTTCCC<br>TACACGACGCTCTTCCGATCTTGTG<br>GAAAGGACGAGGTACCG	PCR
TKO library inner-PCR primer trueseq i5 2	AATGATACGGCGACCACCGAGATC<br>TACACATAGAGGCACACTCTTTCC<br>CTACACGACGCTCTTCCGATCTTGT<br>GGAAAGGACGAGGTACCG	PCR

TKO library inner-PCR primer trueseq i5 3	AATGATACGGCGACCACCGAGATCTACACCCTATCCTACACTCTTTCCCTACACGACGCTCTTCCGATCTTGTGGAAAGGACGAGGTACCG	PCR
TKO library inner-PCR primer trueseq i5 4	AATGATACGGCGACCACCGAGATCTACACGGCTCTGAACACTCTTTCCCTACACGACGCTCTTCCGATCTTGTGGAAAGGACGAGGTACCG	PCR
TKO library inner-PCR primer trueseq i7 1	CAAGCAGAAGACGGCATACGAGATCGAGTAATGTGACTGGAGTTCAGACGTGTGCTCTTCCGATCTATTTTAACTTGCTATTTCTAGCTCTAAAAC	PCR
TKO library inner-PCR primer trueseq i7 2	CAAGCAGAAGACGGCATACGAGATTCTCCGGAGTGACTGGAGTTCAGACGTGTGCTCTTCCGATCTATTTTAACTTGCTATTTCTAGCTCTAAAAC	PCR
TKO library inner-PCR primer trueseq i7 3	CAAGCAGAAGACGGCATACGAGATAATGAGCGGTGACTGGAGTTCAGACGTGTGCTCTTCCGATCTATTTTAACTTGCTATTTCTAGCTCTAAAAC	PCR
TKO library inner-PCR primer trueseq i7 4	CAAGCAGAAGACGGCATACGAGATGGAATCTCGTGACTGGAGTTCAGACGTGTGCTCTTCCGATCTATTTTAACTTGCTATTTCTAGCTCTAAAAC	PCR
TKO library inner-PCR primer trueseq i7 5	CAAGCAGAAGACGGCATACGAGATTTCTGAATGTGACTGGAGTTCAGACGTGTGCTCTTCCGATCTATTTTAACTTGCTATTTCTAGCTCTAAAAC	PCR
TKO library inner-PCR primer trueseq i7 6	CAAGCAGAAGACGGCATACGAGATACGAATTCGTGACTGGAGTTCAGACGTGTGCTCTTCCGATCTATTTTAACTTGCTATTTCTAGCTCTAAAAC	PCR
FIRRM FL attb1 cloning FW	GGGGACAAGTTTGTACAAAAAAGCAGGCTTCATGTTTTTACCTCATATGAACCACC	PCR
FIRRM FL attb1 cloning RV	GGGGACCACTTTGTACAAGAAAGCTGGGTTTCACCCTAGAGTATGTATGTAACGT	PCR

FIRRM NTD quickchange oligo FW	GGGGACAAGTTTGTACAAAAAAGCAGG CTTCATG CATGCATTTCATGCCAATACT TGGA	PCR
FIRRM NTD quickchange oligo RV	GGGGACCACTTTGTACAAGAAAGCTGG GTTTCACCCTAGAGTATGTATGTAACGT	PCR
FIRRM ΔCTD quickchange oligo FW	GTTCGCTGAGGGAACAAATCATGAAGA GATATAGCCATAGTGTCTCAGTTCTGA	PCR
FIRRM ΔCTD quickchange oligo RV	TCAGAACTGAGACACTATGGCTATATC TCTTCATGATTTGTTCCCTCAGCGAAC	PCR
FIRRM ΔWCF quickchange oligo FW	TTTGTTAGCTATGGATGCACTTGCTCGA TATGGGACTG	PCR
FIRRM ΔWCF quickchange oligo RV	CAGTCCCATATCGAGCAAGTGCATCCAT AGCTAACAAA	PCR
FIGNL1 1-120 & 1-360 oligo FW	GGGGACAAGTTTGTACAAAAAAGCAGG CTCCATGCAGACCTCCAGCTCTA	PCR
FIGNL1 1-120 oligo RV	GGGGACCACTTTGTACAAGAAAGCTGGG TCCTATTGCATCATCTTCTGTACACTACTC	PCR
FIGNL1 1-360 oligo RV	GGGGACCACTTTGTACAAGAAAGCTGGG TCCTAAGGCTTACATTGCATTCCT	PCR
FIGNL1 361-674 oligo FW	GGGGACCACTTTGTACAAGAAAGCTGGG TCCTATTGCATCATCTTCTGTACACTACTC	PCR
FIGNL1 121-674 oligo FW	GGGGACAAGTTTGTACAAAAAAGCAGGC TCCATGGCTGGCAAAAAATTCAAAGA	PCR
FIGNL1 361-674 & 121-674 oligo RV	GGGGACCACTTTGTACAAGAAAGCTGGG TCTTACTTTCCACAACCAAAAGTTTT	PCR
FIGNL1 FRBD aa oligo FW	GGGGACAAGTTTGTACAAAAAAGCAGGC TCCATGTTTAAAACTGCAAAAGAACAATT AT	PCR
FIGNL1 FRBD oligo RV	GGGGACCACTTTGTACAAGAAAGCTGGG TCCTATATAGGAGGAACAAACTTTCC	PCR
FIGNL1 ΔFRBD oligo FW	CCCAAGCAAGATGGGGGAGA	PCR
FIGNL1 ΔFRBD oligo RV	TGTAGGCAGGCTGCTATCCTCC	PCR
FIGNL1 F295E oligo FW	CCTACAGAGAAAACTGCAAAAGAAC	PCR
FIGNL1 F295E oligo RV	CAGGCTGCTATCCTCCTTTG	PCR
FIGNL1 K447A oligo FW	GGTGCAACTCTAATTGGCAAG	PCR

FIGNL1 K447A oligo RV	AGTCCCAGGAGGACCAAAG	PCR
FIGNL1 D500A oligo FW	ATTGCCGAAATTGATTCCTTG	PCR
FIGNL1 D500A oligo RV	AAATATCACAGCTGGTTGCTG	PCR
Insect_GFP_fwd	GCTTTCGAATCTAGAGCC	PCR
Insect_GFP_rev	GGCGCCCTGAAAATACAG	PCR
FIGNL1 pFastBac SalI oligo FW	ATTGTCGACCAGACCAGCAGCAGCCGTAGC	PCR
FIGNL1 pFastBac NotI oligo RV	ATTGCGGCCGCTTTGCCGCAACCAAAGGTCTT	PCR
FIGNL1 pGEX SmaI oligo FW	ATTCCCGGGCAGACCAGCAGCAGCCGTAGC	PCR
FIGNL1 pGEX XhoI oligo RV	ATTCTCGAGTTTGCCGCAACCAAAGGTCTT	PCR
FIRRM pFastBac SalI oligo FW	ATTGTCGACGTCATGTTTCTGCCGCACATG	PCR
FIRRM pFastBac NotI oligo RV	ATTGCGGCCGCACCCAGGGTGTGGATGTAACG	PCR
FIRRM pGEX BamHI oligo FW	ATTGGATCCGTCATGTTTCTGCCGCACATG	PCR
FIRRM pGEX XhoI oligo RV	ATTCTCGAGACCCAGGGTGTGGATGTAACG	PCR
FIGNL1c.o._fwd	ACCTGTATTTTCAGGGCGCCATGCAGACCTCCAGCTCTAG	PCR
FIGNL1c.o._rev	CAGGCTCTAGATTCGAAAGCTTACTTTCCACAACCAAAAGTTTTG	PCR
FIRRMc.o._fwd	ATTGCCGAAATTGATTCCTTG	PCR
FIRRMc.o._rev	AAATATCACAGCTGGTTGCTG	PCR
FAM-labeled ssDNA	5’-FAM-GACGCTGCCGAATTCTACCAGTGCCTTGCTAGGACATCTTTGCCCACCTGCAGGTTCACC-3’	EMSA-UnFix
SA-labeled	5’-FAM-GCCACTGCCGTCGTCTGCCACACCGCTCGGTTCACATCTATGAACACTTGCAGGTTCACT-3’	EMSA-UnFix
SA-unlabeled	5’-CTAATCTTGAGCTAACAGGGATATGTCCTTCCGAGCGGTGTGGCAGACGACGGCAGTGGC-3’	EMSA-UnFix
JYM 925	GGGTGAACCTGCAGGTGGGCAAAGATGTCCTGCAATGTAATCGTCAAGCTTTATGCCGT	EMSA-FIX
JYM 926	ACGCTGCCGAATTCTACCAGTGCCAGCGACGGACATCTTTGCCCACCTGCAGGTTCACCC	EMSA-FIX

JYM 945	ACGGCATAAAGCTTGACGATTACATTGCTAGGA CATCTTTGCCCACCTGCAGGTTCACCC	EMSA-FIX
JYM 1086	5’ biotin - [T]83	EMSA-FIX
FIGNL1 siRNA resistant mutagenesis FW	GAGTCATCTAGAAGGATAAAAACAGAA	PCR
FIGNL1 siRNA resistant mutagenesis RV	GTGTTCACCATCTCCCCGTT	PCR

[a] KD: knockdown

Table S5. Antibodies used in this study.

**Primary antibodies**		
**Target**	**Catalog number**	**Used for**
Rabbit anti-53BP1	Novus Cat# NB100-304, RRID:AB_10003037	IF
Rabbit anti-BRCA1	Millipore Cat# 07-434, RRID:AB_2275035	IF
Mouse anti-BRCA2	Millipore Cat# OP95, RRID:AB_2067762	IF
Human anti-centromere CREST	Erba Diagnostics Cat# HCT-0100, RRID:AB_2744669	IF
Rabbit anti-mCherry	Novus Cat# NBP2-25157, RRID:AB_2753204	WB
Mouse anti-Cyclin A1	BD Biosciences Cat# 611269, RRID:AB_398797	IF
Rabbit anti-FIRRM	Abcam Cat# ab121774, RRID:AB_11132686	WB, IP
Rabbit anti-FANCA	Thermo Fisher Scientific Cat# A301-980A-M, RRID:AB_2780254	WB
Rabbit anti-FANCD2	Novus Cat# NB100-182, RRID:AB_10002867	IF
Mouse anti-FLAG	Sigma-Aldrich Cat# F3165, RRID:AB_259529	WB, IP
Mouse anti-GFP IgG1κ clones 7.1 and 13.1	Sigma-Aldrich Cat# 11814460001, RRID:AB_390913	WB
Mouse anti-phospho-Histone H2A.X Ser139 (γ-H2AX)	Millipore Cat# 05-636, RRID:AB_309864	IF
Rabbit anti-Lamin-B	Abcam Cat# ab16048, RRID:AB_443298	IF
Rabbit anti-RAD51	Abcam Cat# ab16048, RRID:AB_443298	IF
Mouse anti-RAD51 14B4	Novus Cat# NB100-148, RRID:AB_10002131	IP
Anti-Streptavidin HRP conjugated	GE Healhcare Cat# RPN1231V	WB
Mouse anti-α-Tubulin	Sigma-Aldrich Cat# CP06, RRID:AB_2617116	WB
Mouse anti-BrdU	Cytiva Cat# RPN 202, RRID:AB_2314032	IF
Rabbit anti-PCNA	Novus Cat# NBP2-67390	IF
**Secondary antibodies**		
**Target**	**Catalog number**	**Used for**
Alexa Fluor 488 goat anti-rabbit	Thermo Fisher Scientific Cat# A-11034, RRID:AB_2576217	IF

Alexa Fluor 555 goat anti-rabbit	Molecular Probes Cat# A-21428, RRID:AB_141784	IF
Alexa Fluor 647 goat anti-rabbit	Molecular Probes Cat# A-21244, RRID:AB_2535812	IF
Alexa Fluor 488 goat anti-mouse	Thermo Fisher Scientific Cat# A-11029, RRID:AB_2534088	IF
Alexa Fluor 555 goat anti-mouse	Thermo Fisher Scientific Cat# A-21424, RRID:AB_141780	IF
Alexa Fluor 647 goat anti-mouse	Thermo Fisher Scientific Cat# A-21236, RRID:AB_2535805	IF
Alexa Fluor 647 donkey anti-human	Jackson ImmunoResearch Labs Cat# 709-605-098, RRID:AB_2340577	IF
Goat anti-rabbit HRP	Jackson ImmunoResearch Labs Cat# 111-035-144, RRID:AB_2307391	WB
Sheep anti-mouse-HRP	Sigma-Aldrich Cat# A9044, RRID:AB_258431	WB

Table S6. Plasmids used in this study.

Plasmid	Source
lentiCas9-Blast	Addgene #52962
Toronto KnockOut TKO CRISPR library – v1	Addgene #1000000069
pKLV2-U6gRNA5BbsI-PGKpuro2AmCherry	Addgene #67977
pKLV2-U6gRNA5BbsI-PGKpuro2ABFP	Addgene #67991
pOG44	Invitrogen^{TM} V600520
pDEST-pcDNA5-FLAG-miniturbo	This study
FLAG-miniturbo-FIRRM	This study
pDEST-pcDNA5-FRT-TO-mCherry-LacRnls	Described in *(59)*
mCherry-LacRnls FIRRM FL 1-854	This study
mCherry-LacRnls FIGNL1 1-674	This study
mCherry-LacRnls FIGNL1 1-120	This study
mCherry-LacRnls FIGNL1 121-674	This study
mCherry-LacRnls FIGNL1 1-360	This study
mCherry-LacRnls FIGNL1 361-674	This study
mCherry-LacRnls FIGNL1 FRBD 295-344	This study
mCherry-LacRnls FIGNL1 ΔFRBD Δ295-344	This study
mCherry-LacRnls FIGNL1 F295E	This study
mCherry-LacRnls FIGNL1 K447A	This study
mCherry-LacRnls FIGNL1 D500A	This study
mCherry-LacRnls FIGNL1 KDm K447A/D500A	This study
pDEST-pcDNA5-FRT-TO-eGFP	Described in *(14)*
GFP-FIRRM FL 1-854	This study
GFP-FIRRM NTD 1-175	This study
GFP-FIRRM ΔCTD 1-735	This study
GFP-FIRRM ΔWCF 461-463	This study
GFP-FIGNL1 FL 1-674	This study
pDEST-pCW57.1-TurboID-HA-2xStrep	Described in *(61)*
TurboID-HA-2xStrep-FIRRM FL 1-854	This study
TurboID-HA-2xStrep-ΔWCF 461-463	This study
pDEST-pCW57.1-GFP	Described in *(59)*
GFPnls-FIRRM FL 1-854	This study
GFPnls-FIRRM ΔWCF 461-463	This study
pCBASceI	Addgene #26477
pGEX-6P-2-GST	GE Healthcare #28-9546-50
GST-FIRRM-His	This study
GST-FIGNL1-His	This study
pFastBac dual vector	Invitrogen #10712024
GFP-TEV-FIRRM	This study
GFP-TEV-FIGNL1	This study
pDEST-pcDNA5-FRT-TO-3xFlag	Described in *(14)*
3xFlag-FIRRM FL 1-854	This study
3xFlag-ΔWCF 461-463	This study
